# Ecofriendly solidification of sand using microbially induced calcium phosphate precipitation

**DOI:** 10.1038/s41598-024-63016-9

**Published:** 2024-05-30

**Authors:** Maksym Avramenko, Kazunori Nakashima, Chikara Takano, Satoru Kawasaki

**Affiliations:** 1 Graduate School of Engineering, Hokkaido University, Sapporo, Japan; 2 Faculty of Engineering, Hokkaido University, Sapporo, Japan

**Keywords:** Soil improvement, Microbial induced calcium phosphate precipitation, Calcium phosphate compounds, Tuna fish bones, Lactic acid bacteria, Biogeochemistry, Environmental sciences

## Abstract

This study introduces microbiologically induced calcium phosphate precipitation (MICPP) as a novel and environmentally sustainable method of soil stabilization. Using *Limosilactobacillus sp.*, especially NBRC 14511 and fish bone solution (FBS) extracted from Tuna fish bones, the study was aimed at testing the feasibility of calcium phosphate compounds (CPCs) deposition and sand stabilization. Dynamic changes in pH and calcium ion (Ca^2+^) concentration during the precipitation experiments affected the precipitation and sequential conversion of dicalcium phosphate dihydrate (DCPD) to hydroxyapatite (HAp), which was confirmed by XRD and SEM analysis. Sand solidification experiments demonstrated improvements in unconfined compressive strength (UCS), especially at higher Urea/Ca^2+^ ratios. The UCS values obtained were 10.35 MPa at a ratio of 2.0, 3.34 MPa at a ratio of 1.0, and 0.43 MPa at a ratio of 0.5, highlighting the advantages of MICPP over traditional methods. Microstructural analysis further clarified the mineral composition, demonstrating the potential of MICPP in environmentally friendly soil engineering. The study highlights the promise of MICPP for sustainable soil stabilization, offering improved mechanical properties and reducing environmental impact, paving the way for novel geotechnical practices.

## Introduction

The current global trend toward sustainable engineering practices has become essential, driven by the need to achieve zero emissions by 2050 in response to rising carbon dioxide levels^1^. This trend has its foundation in the collective determination of the 195 countries that ratified the Paris Agreement, committing to intensify efforts to reduce emissions to mitigate the effects of climate change^2^. Core to this obligation is a focus on keeping global warming to well below 2 °C compared to pre-industrial levels, and a concerted effort to limit temperature rise to 1.5 °C^3^. However, an assessment of recent nationally determined contributions and public statements by key global players, including the United States, China, the EU, and Japan, which together account for 56% of global greenhouse gas emissions, indicates that the necessary measures to mitigate global warming are not sufficient. As a result, the current global warming scenarios predict a temperature increase of between 1.8 and 2.4 °C over this century^4^. Considering these findings, it is imperative for the global community to intensify efforts toward sustainability, with particular emphasis on the construction sector, which accounts for 30% of total greenhouse emissions^5^.

Over the past two decades, the field of biomineralization has advanced significantly, offering a potentially environmentally friendly technology with diverse applications in engineering^6^. Biomineralization is the process by which living organisms produce minerals, such as calcium carbonate, as a byproduct of their metabolic processes. This phenomenon has garnered increasing interest from researchers due to its potential applications in materials science, environmental biotechnology, and medicine^7^.

The applications of biomineralization are extensive and varied, encompassing the development of biological building products capable of replacing energy-intensive materials like cement. Moreover, biomineralization holds promise in surface and near-surface stabilization applications, which can enhance soil stability and mitigate toxic chemical contamination^8^. Moreover, biominerals have demonstrated potential in serving as a biocement or assisting in sealing cracks in self-healing Portland cement concrete. This application can enhance concrete strength and reduce water absorption^9,10^.

One particularly promising method within biomineralization is microbially induced carbonate precipitation (MICP). MICP involves the precipitation of calcite by microorganisms through a urease reaction. Numerous studies have focused on its application in soil stabilization, soil strength enhancement, and bioremediation^11–13^, making it an innovative and sustainable technology with wide-ranging applications in geotechnical, hydraulic, geological, ocean, and environmental engineering^14^.

Furthermore, MICP is emerging as a critical component of Carbon Capture and Storage efforts aimed at reducing CO_2_ emissions. By capturing carbon dioxide from industrial facilities and power plants and storing it underground or converting it into a useful form, MICP technology offers environmental benefits^15^. Futhermore, compared to traditional engineering methods, MICP has been found to have less physical impact on the environment^14^.

However, MICP is not without its drawbacks. One major concern is the production of urea hydrolysis byproducts, namely ammonia and ammonium, which can have harmful effects on human health, vegetation, and contribute to atmospheric nitrogen deposition^16^. Ammonium ions exhibit a reduced susceptibility to soil leaching compared to gaseous ammonia, facilitated by their capacity to bind with negatively charged soil colloids and displace other cations. The challenges posed by ammonia volatilization have prompted the establishment of international objectives and regulations aimed at mitigating anthropogenic ammonia emissions, such as the Gothenburg Protocol^17^. These emissions have posed a significant constraint on the widespread adoption of soil strengthening techniques based on MICP, sparking debates regarding the true environmental sustainability of this approach^18^.

To address environmental concerns associated with MICP, a novel eco-friendly method has been proposed. This method involves urea hydrolysis at low pH to precipitate calcium phosphate compounds (CPCs), thereby preventing the release of gaseous ammonia. The decrease in pH of the environment leads to a reduction in the amount of ammonia converted from ammonium, which subsequently decreases its release into the atmosphere^19^.

The precipitation of CPCs necessitates an increase in pH in a medium rich in calcium and phosphorus. While numerous studies have explored the controlled precipitation of CPCs using various chemicals, such as diammonium phosphate, dipotassium phosphate, calcium acetate, and calcium nitrate mixed with different phosphate and carbonate powders^20^, there remains a gap in research on the deposition of CPCs for soil improvement using natural resources^19^.

In the realm of engineering applications, several innovative approaches have been utilized thus far. These include employing acidotolerant urease-producing bacteria and bone meal for repairing cracked stone^21^, as well as applying dimorphic phytase-active yeast and calcium phytate solution to precipitate various CPCs^22^. Notably, our prior research showcased the effectiveness of utilizing acid urease and Tuna fish bone solution to improve the quality of both fine and coarse sand^19^.

While CPCs are soluble in acids and rapidly precipitate in alkaline solutions, finding applications in medicine, such as coatings for metal implants and synthetic bone substitutes, has proven their environmental harmlessness^23,24^. However, the application of CPCs for soil improvement represents a novel approach in the engineering field, with limited research conducted thus far^20^.

In this study, Tuna fish bones are used as an eco-friendly source of calcium and phosphorus, which are often considered waste products in Tuna production^25^. Among different types of fish bones, Tuna bones exhibit superior concentrations of calcium and phosphorus ions^26–30^ surpassing those found in bones from cattle, chickens, and pigs^31^. Given the global prominence of Tuna consumption, with Tuna being the most widely consumed fish worldwide^32^, it consequently generates a substantial volume of waste post-production. Utilizing Tuna fish bones in the MICPP method not only reduces waste generation but also promotes circularity within the food industry, aligning with Sustainable Development Goals (SDGs). Strains of lactic acid bacteria (LAB), known for their environmental harmlessness and widespread use in food industry, are used to increase pH in the acidic range^33^.

This study presents a novel, environmentally friendly soil improvement method that examines the impact of Tuna fish bones as a source of calcium and phosphorus in the CPCs precipitation process through MICPP reaction. The study evaluates the effectiveness of LAB during solidification by applying different urea ratios and testing the strength of fine sand samples over time. Before testing, the bacterial growth, urease activity, and ability to precipitate CPCs over time were evaluated. The sediment and compacted soil samples were tested using SEM, SEM–EDS and XRD to confirm the deposition of CPCs and determine its origin. Additionally, quantitative analysis of UCS and precipitation content was evaluated to provide optimal formulations for future studies.

## Materials and methods

### Materials

#### Sand

To explore the influence of bacterial activity on calcium phosphate precipitation efficiency within the framework of the MICPP method, Toyoura sand was obtained from Toyoura Keiseki Kogyo Co., Ltd. Well-known for its popularity in geotechnical research, this natural silica sand is characterized by rounded particles and exceptional fluidity, ensuring consistent and stable outcomes when utilized in experiments^34^. The silica sand, primarily composed of SiO_2_, is well-regarded for its reliability in producing uniform and accurate results.

**Figure 1:**
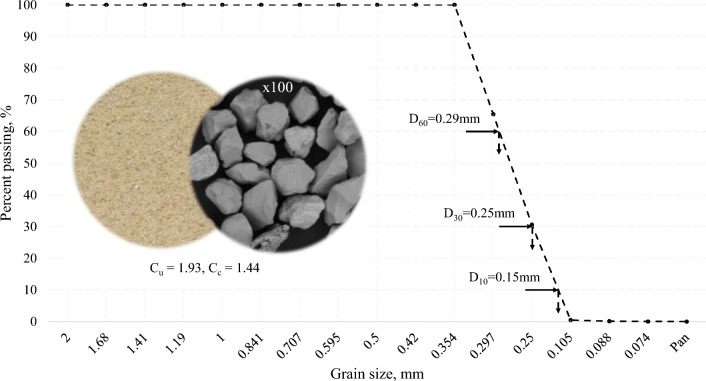
Grain size distribution curve of Toyoura sand. Note: C_u_—Coefficient of uniformity, C_c_—Coefficient of curvature.

Following the acquisition of the Toyoura sand, a testing protocol was carried out. The particle size distribution analysis was carried out in accordance with ASTM D6913-04^35^, and the results are presented in Fig. 1. The detailed analysis of particle size distribution provides a solid foundation for further research into the suitability of Toyoura sand for a targeted study. The sand is classified as poorly graded (SP)^36^.

#### Fish bone cementation solution

**Figure 2:**
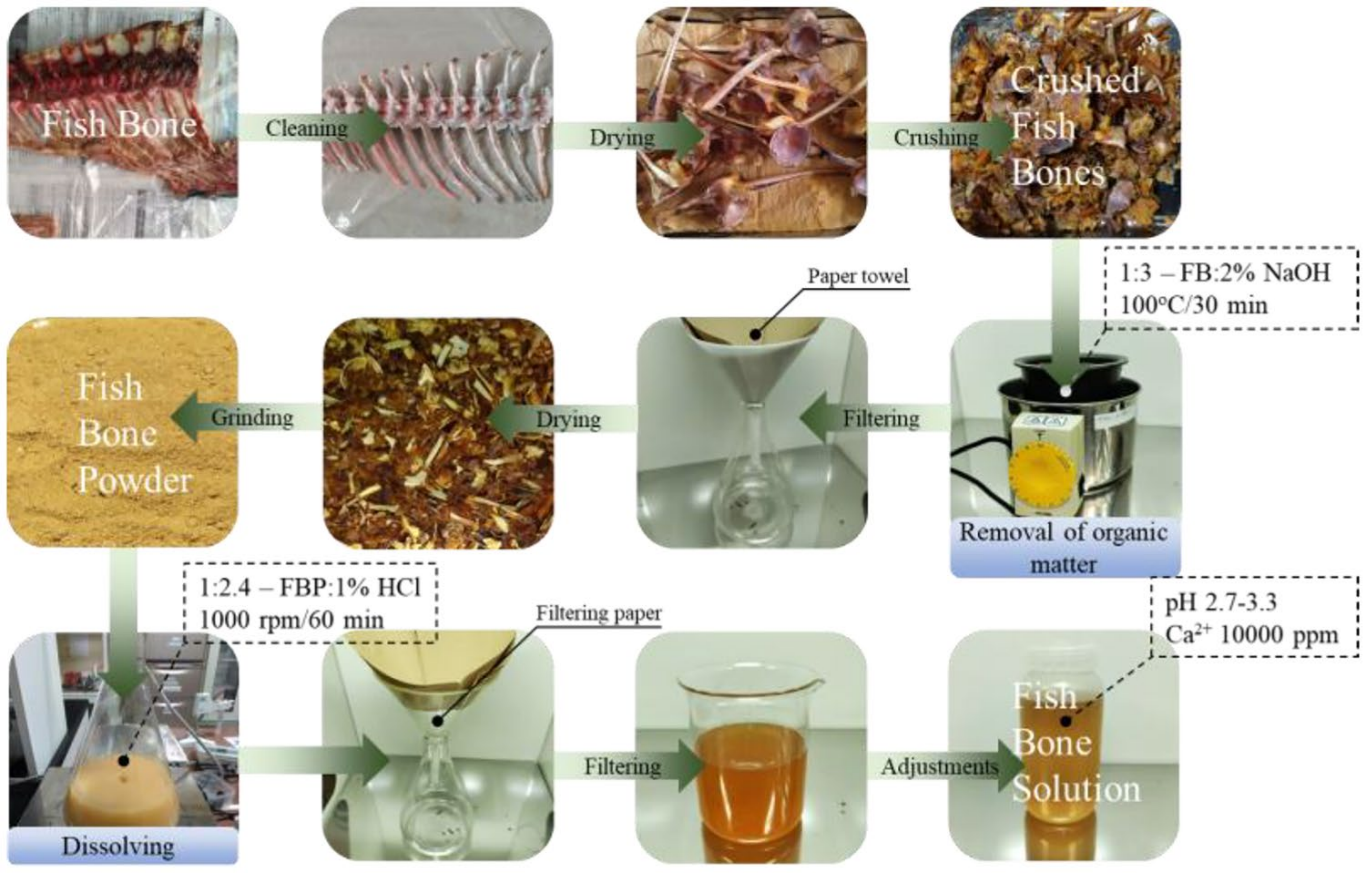
Flowchart of fish bone solution preparation.

In this study, fish bone solution (FBS) served as the primary source of calcium and phosphorus for the precipitation of CPCs via the MICPP method. The alkaline method outlined by^37^ for preparing Tuna fish bone solution was employed with certain modifications to extract calcium and phosphorus from fish bones. A detailed schematic representation of the FBS preparation process is presented in Fig. 2.

Tuna bones, which were obtained as waste with the remaining flesh, were provided by a local supplier. The residual flesh was manually removed, and the bones were segmented to accelerate the drying process. Following a 48-h period at room temperature, the bone segments were ground into pieces approximately 1–2 cm in size and subsequently boiled in a 2% NaOH solution for 30 min (1:3 w/v) to remove of organic matter. The resulting solution underwent filtration using a paper towel, and the fish bones were left to dry under room temperature conditions. Once completely dried, the fish bones were further crushed into smaller pieces and ground into a powder with particle sizes less than 1 mm.

The dried and ground fish bone powder was dissolved in a 1% HCl solution (1:2.4 w/v) through stirring for 60 min at 1000 rpm. Hydrochloric acid was utilized in the process to extract calcium and phosphorus ions from the fish bones, as represented by Eq. (1). The solution was then filtered, and the pH and calcium ion concentration were adjusted to be within the range of pH 2.7–3.3 and 10,000 ppm, respectively.

1 $${\text{Ca}}_{10} \left( {{\text{PO}}_{4} } \right)_{6} \left( {{\text{OH}}} \right)_{2} + 8{\text{HCl}} \to 10{\text{Ca}}^{2 + } + 6{\text{HPO}}_{4}^{2 - } + 8{\text{Cl}}^{ - } + 2{\text{H}}_{2} {\text{O}}$$

#### Bacteria, culture medium and urea

The bacterial strains investigated in this study belonged to the *Limosilactobacillus sp.*, specifically strains NBRC 14511, 14512, and 14513, generously provided by the Biological Resource Center, National Institute of Technology and Evaluation, Tokyo, Japan. Upon retrieval of the bacterial cultures from their respective ampules, rehydration was initiated by introducing the bacteria into a medium composed of Hipolypepton (10 g), Yeast extract (2 g), and MgSO_4_·7H_2_O (1 g) dissolved in 1 L of distilled water. Subsequently, after mixing the rehydration medium with the bacterial culture, the resulting bacterial solution was inoculated onto agar plates supplemented with Hipolypepton (5 g), Yeast extract (5 g), Glucose (5 g), MgSO_4_·7H_2_O (1 g), and Agar (15 g). Incubation of these plates at 37 °C within containers equipped with anaerobic bags facilitated the establishment of anaerobic conditions conducive to bacterial growth.

After successful propagation of the bacteria on rehydration agar plates, the samples were transferred to Man-Rogosa-Sharpe (MRS) medium, a standard nutrient medium widely used for cultivation of LAB^38^, consisting of MRS Broth at 55 g dissolved in 1 L of distilled water. These cultures served as the basis for subsequent experimental analyses.

In the experimental procedures, commercially available synthetic urea obtained from FUJIFILM Wako Pure Chemical Corporation, Osaka, Japan, was employed. This synthetic urea served as a vital nitrogen source for LAB and played a pivotal role in inducing localized pH elevation through urea hydrolysis.

### Methods

#### Measurements

During the experiments, pH and Ca^2+^ concentrations were carefully measured. The pH measurements were conducted using a pen-type pH meter (pH5S, CEM Corporation, Tokyo, Japan), while the calcium ion concentration in the solution was quantified using a water quality meter (LAQUAtwin Ca-11, HORIBA Advanced Techno Corporation, Kyoto, Japan).

Ammonium concentrations were assessed utilizing the Ammonia High Range Photometer HI97733 (Hanna Instruments Inc., Woonsocket, USA). The instrument employs a sensitive probe that reacts specifically with ammonium ions in the water, producing a measurable signal. The measurements were taken in triplicate to ensure precision.

To assess urease activity in the bacteria, the indophenol-blue method was employed as described in previous research^39^. Spectrophotometric analysis, utilizing a V-730 spectrophotometer (JASCO Corporation, Tokyo, Japan), was employed to ascertain urease activity values. Urease activity values were determined by establishing calibration curves correlating different ammonium ion concentrations with intensities OD_630_.

For quantifying bacterial cells concentration, optical density (OD_600_) was measured using a spectrophotometer (V-730) at a wavelength of 600 nm. Each sample was measured and compared with the OD_600_ of the control MRS medium without bacteria. The difference between OD_600_ measurements was utilized to represent the cell density in this paper.

UCS of the specimens was determined through needle penetration tests using the SH-70 apparatus (Maruto Testing Machine Company, Tokyo, Japan), adhering to the standard protocol outlined by the Japanese Geotechnical Society (JGS 0511-2009, Method for unconfined compression test of soils)^40^.

A needle penetrometer is a nondestructive testing device developed for geotechnical research in Japan, is utilized to assess the strength of soft rocks and stabilized soil. The needle penetrometer eliminates the need for sample preparation and can be employed in both field and laboratory settings with minimal surface preparation. The needle penetration test demonstrates reliability in predicting UCS for solidified sand and similar materials with UCS values of up to 30 MPa^41^.

**Figure 3:**
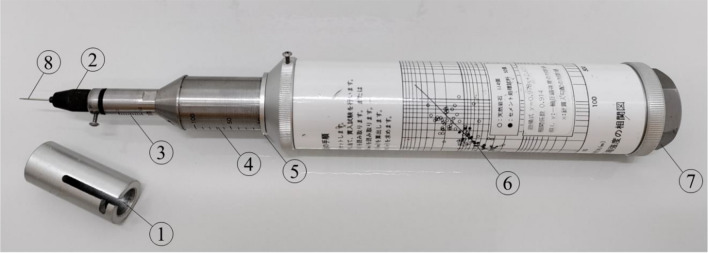
Maruto SH-70 Needle Penetrometer.

Figure 3 illustrates the visual representation of the Maruto SH-70 apparatus along with its principal components. Before conducting the experiment, the presser (1) is to be positioned onto the chuck (2) with the penetration needle (8) securely inserted and fixed. Once the device is placed at the desired location for assessing the UCS, it should be loaded with force, and the results can be observed on both the penetration scale (3) and the load scale (4) using the load indicating ring (5). Upon completion of the experiment, the load and penetration depth results are to be calculated into UCS utilizing the correlation diagram (6). The penetration needle is then to be carefully placed inside the designated area beneath the removable cap (7).

In this study, samples that were completely dried underwent horizontal examination for UCS using the Maruto SH-70 device at three distinct locations: Top, Middle, and Bottom. The Top location was defined as being 1cm from the top surface of the sample, the Middle location was at the midpoint of the sample, and the Bottom location was situated 1cm from the bottom surface. Penetration and load scale results were obtained from these locations, and subsequently, UCS values (expressed in kilopascals, kPa) were calculated utilizing Eq. (2).

2 $$\log_{10} \;{\text{UCS}} = 0.978\log_{10} \frac{{{\text{Load}}\;{\text{ scale}}\;{\text{ result}}}}{{{\text{Penetration}}\; {\text{scale}}\;{\text{ result}}}} + 2.621$$

The SEM and SEM–EDS were performed using a Scanning Electron Microscope (JSM-IT200, JEOL Ltd., Tokyo, Japan) with an accelerating voltage of 15kV. Prior to analysis, each sample was preliminarily coated with gold using a Sputter Coater (DII-29010SCTR Smart Coater, JEOL Ltd., Tokyo, Japan) for a duration of 2 min.

To obtain XRD results of the precipitated minerals, the enhanced soil sample was subjected to mineralogical composition analysis using a MultiFlex X-ray diffractometer (Rigaku Corporation, Tokyo, Japan). The instrument operated with a Cu K-alpha source, a scan rate of 6.5°/min, and a diffraction angle 2θ value ranging from 5° to 60°.

Precipitation content was assessed through an acid reaction by mixing 5ml of 1% HCl with 1g of cemented sand. After 24 h samples were rinsed with distilled water and dried in 60 °C oven. The mass difference of dry samples before and after the acid reaction was calculated as a percentage (Eq. 3).

3 $${\text{Precipitation}}\;{\text{ content}}\; \left( \% \right) = \frac{{{\text{Dry}}\;{\text{ mass}}\;{\text{ before}}\;{\text{ acid}}\;{\text{ reaction}} - {\text{Dry}}\;{\text{ mass}}\;{\text{ after}}\;{\text{ acid}}\;{\text{ reaction}}}}{{{\text{Dry}}\;{\text{ mass}}\;{\text{ before}}\;{\text{ acid}}\;{\text{ reaction}}}} \times 100\%$$

#### Testing methods for bacteria

After successful rehydration and cultivation of the bacteria, the bacteria were used in a pre-cultivation medium consisting of 5 ml of MRS medium. The bacterial samples were placed in an incubator at 37 °C for 24 h under anaerobic conditions. Subsequently, 1 ml of the pre-culture was transferred to 50 ml of MRS medium, designating the start time as 0 h. The concentration of urease cells was measured at 0, 1, 3, 6, and 12 h, followed by daily measurements until day 7 with daily assessment of urease activity.

To measure urease activity in the bacteria, a 100 ml beaker was used to prepare a mixture consisting of 50 ml Citrate buffer with pH adjustments to 3, 4, 5, and 6, along with 0.3 g of Urea. This beaker was then placed in a heated water basin, ensuring constant temperatures were maintained at 25, 30, and 37 °C. After reaching the desired temperature, 0.5 ml of the bacterial sample was added, and samples were collected at 0, 5, 10, and 15 min for assessing urease activity via the indophenol-blue reaction. Following urea hydrolysis, ammonium ions reacted with phenol to produce a blue indigo dye. The intensity of this dye was then measured as described in Measurements part.

#### Precipitation method

To evaluate the performance of the bacteria in a dedicated environment, specifically in an FBS, and to confirm their ability to precipitate CPCs in this environment, a precipitation method was applied. This procedure was carried out to identify and select samples suitable for further solidification experiments.

In this study, LAB were utilized to elevate the pH of the FBS medium. In acidic conditions, the enzymatic hydrolysis of urea by LAB leads to the formation of ammonium ions and carbonate ions, which contributes to an increase in pH, (Eq. 4)^42^. This pH adjustment served to initiate the precipitation of CPCs, and the FBS medium, abundant in calcium and phosphorus, provided the necessary components for CPCs formation.

4 $${\text{CO}}\left( {{\text{NH}}_{2} } \right)_{2} + 2{\text{H}}_{2} {\text{O}}\to ^{{{\text{LAB}}}} 2{\text{NH}}_{4}^{ + } + {\text{CO}}_{3}^{2 - } \left( {{\text{pH}} \uparrow } \right)$$

The precipitation method involved the application of the bacterial strain NBRC 14511, which exhibited urease activity of 1.44 μmol/min/ml after 7 days of incubation in MRS medium at 37 °C. The experimental solution comprised 8 mL of FBS and 2 ml of the bacterial solution (4.34 × 10^9^ bacteria cells/ml). To examine the time dynamics of CPCs deposition in more detail, various time intervals (1, 3, and 7 days) were systematically chosen. This study was complemented by an investigation of different Urea/Ca^2+^ ratios (0.15, 0.25, 0.5, 1.0, 2.0) to identify the optimal composition for further implementation in solidification tests. As a source of urea was chosen commercially available urea. These ratios were chosen based on established practices employed in enzymatic hydrolysis of urea^43^. The precipitation process was conducted at a temperature of 37°C, with a Ca^2+^ concentration of 10,000 ppm, and a FBS pH of 3.

Following the inoculation of the bacterium into the provided medium, pH and Ca^2+^ concentrations were monitored at intervals of 1, 3, 6, 12 h, and subsequently, daily until the completion of the deposition period. After precipitation, the samples were rinsed with distilled water and then dried for 48 h in an oven at 60°C. Subsequently, the precipitate content was estimated using the previously described method and analyzed by SEM, SEM–EDS, and XRD.

#### Sand solidification method

**Figure 4:**
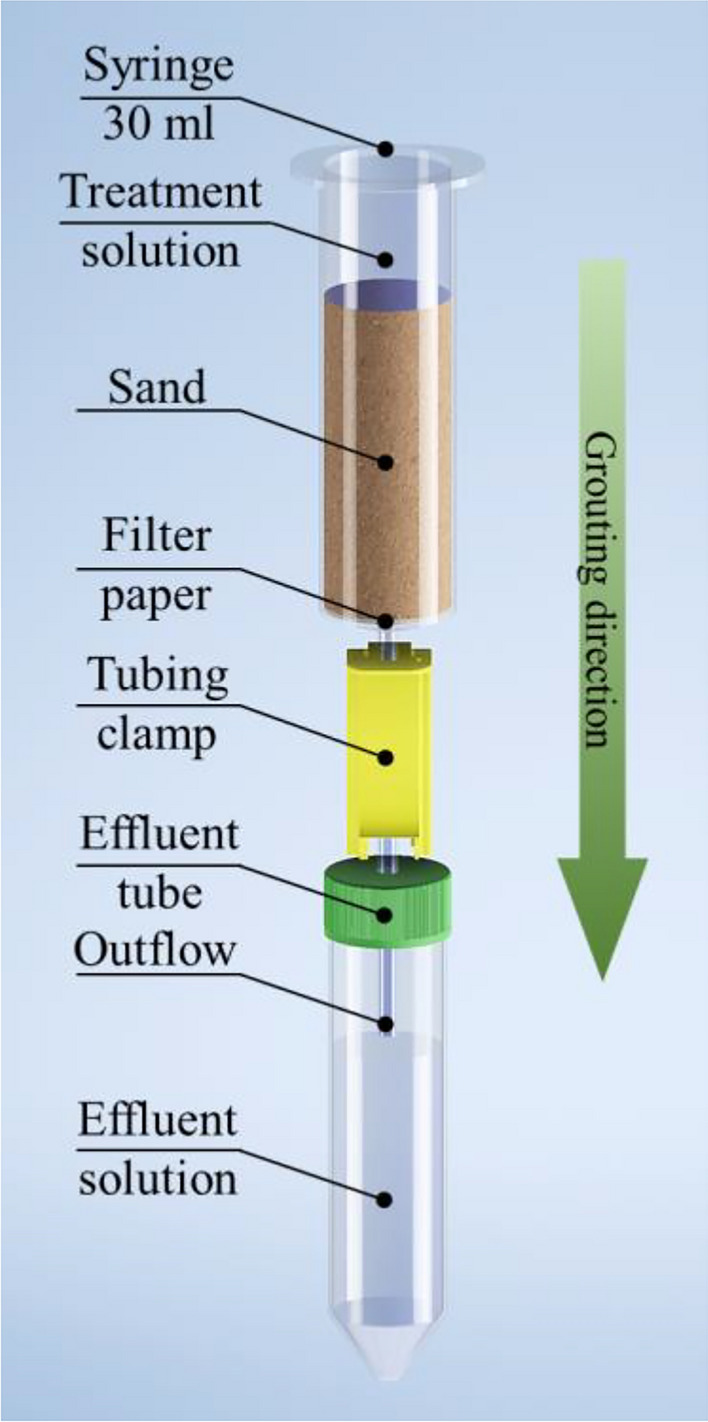
Schematic diagram of sand solidification test.

To solidify the soil samples utilizing MICPP, a thorough experiment was conducted, the details of which are illustrated in Fig. 4. The procedure consists of initially placing filter paper on the bottom of the 30 ml syringe and then filling it with pre-dried Toyoura sand up to the 30 ml mark. This precautionary measure prevents the occurrence of sand leakage during the experiment.

An efficient outflow system was used, connecting the syringe to an effluent tube equipped with a tubing clamp to control the flow of liquid through the sample, which was maintained at approximately 3 ml/min. The experimental protocol lasted 14 days, including daily injections of 10 ml of treatment solution. The treatment solution comprises 10 ml of FBS mixed with varying amounts of urea as per the experimental design. Additionally, 10 ml injections of bacterial solution (4.3 × 10^9^ bacteria cells/ml) were introduced on days 1 and 7 two hours before the injection of the treatment solution to optimize distribution of bacteria in the sample.

The bacterial solution obtained from NBRC 14511 underwent a 7-day incubation in MRS medium at 37 °C, demonstrating urease activity of 1.54 μmol/min/ml and 1.56 μmol/min/ml during the first and second injections, respectively. Urea/Ca^2+^ ratios of 0.5, 1.0, and 2.0 were studied at a constant temperature of 37 °C throughout the experiment. FBS as a treatment solution with a Ca^2+^ concentration of 10,000 ppm and pH3 was used as a rich source of calcium and phosphorus for the precipitation of CPCs.

Throughout the experiment, the pH, calcium, and ammonium ion concentrations in the effluent solution were monitored daily. After the solidification period, 10 ml of distilled water was added to each sample to rinse them and stop the reaction. Subsequently, the separated syringe with the compacted soil sample was placed in a 60°C oven for 48 h to dry thoroughly. Following drying, the cylinder was opened, and the sample was carefully transferred to a tray for UCS testing, precipitation content evaluation, and SEM, SEM–EDS, and XRD analysis.

## Results and discussion

### Biological properties

#### Effect of temperature on urease activity and bacterial growth

**Figure 5:**
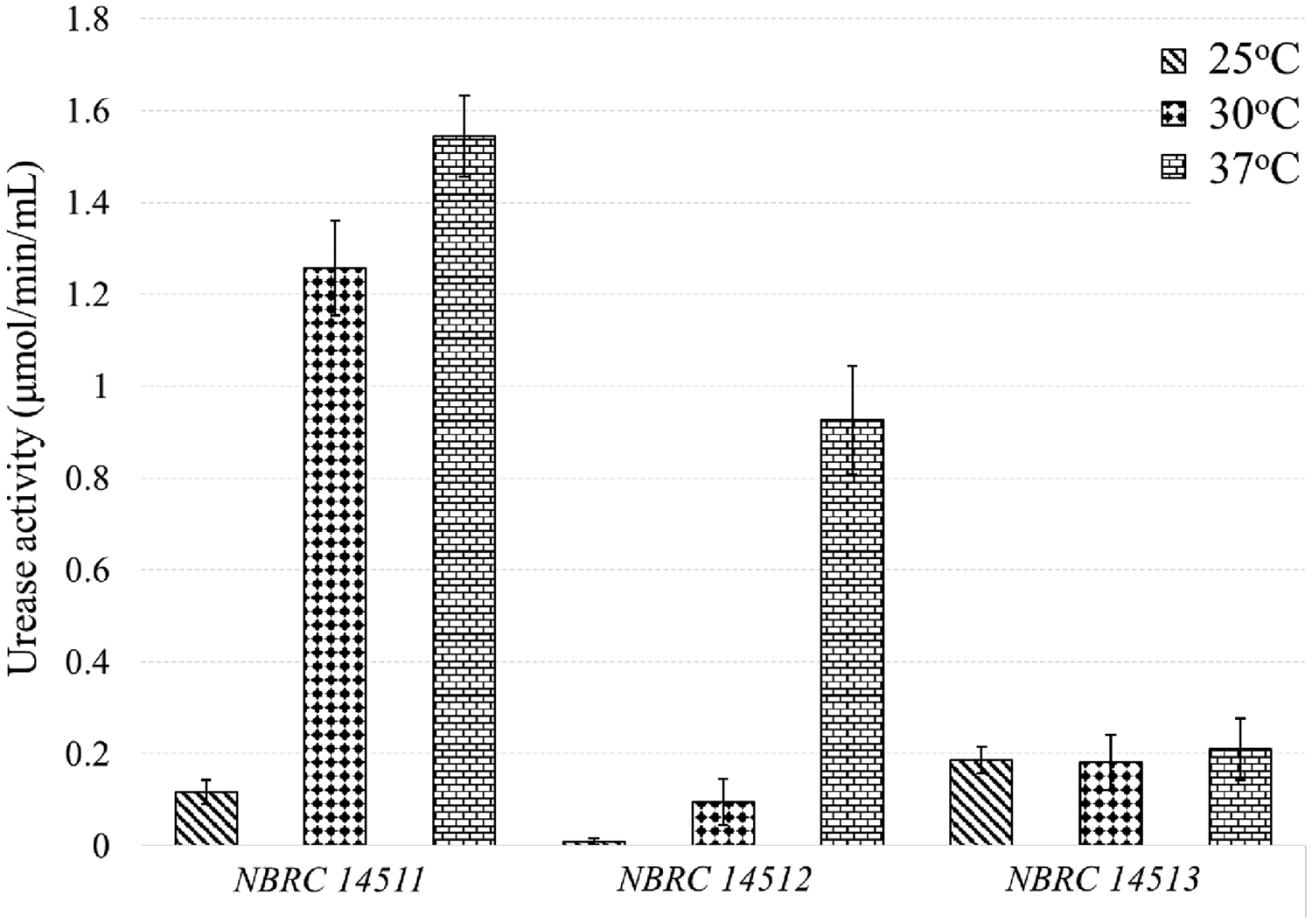
Urease activity test results of all purchased LAB species after 168 h of culture.

Figure 5 illustrates the results of urease activity for the accessible bacterial strains. The unit of activity is operationally defined as the quantity of urea (expressed in micromoles) hydrolyzed per minute by 1 ml of bacterial culture, enriched under the specified experimental conditions. It should be noted that strain NBRC 14511 shows the highest values at 30 and 37 °C, which are 1.24 μmol/min/ml and 1.54 μmol/min/ml, respectively, indicating that it is suitable for further tests, especially at 37 °C. Strain NBRC 14512 shows a similar trend, registering the lowest result at 25 °C and the highest at 37 °C. In contrast, strain NBRC 14513 does not show a peak or significant increase in urease activity, maintaining a relatively low activity at all temperatures tested.

Comparing these findings to previous research, the urease activity of NBRC 14511 aligns with that of *Lactobacillus fermentum* strains, which have been reported to exhibit urease activity ranging from 1.0 to 5.0 μmol/min/ml^44,45^, depending on factors such as pH and experimental conditions*.* Researchers in the field of MICP typically use bacteria with urease activity between 2.7 and 4.0 μmol/min/ml for their experiments^46–49^. However, there are also applications where lower urease activity, such as 1.9^50^, and 1.0 μmol/min/ml^51^, has been reported. The study results indicate that NBRC 14511 exhibits urease activity typical of *Lactobacillus fermentum* strains, with sufficient urease activity. This characteristic makes it suitable for adjusting pH in microbially induced CPCs precipitation.

To investigate the bacterial growth kinetics of the strain with the highest urease activity, the bacterial cell concentration was measured after 1, 3, 6, and 12 h, followed by daily measurements until day 7.

**Figure 6:**
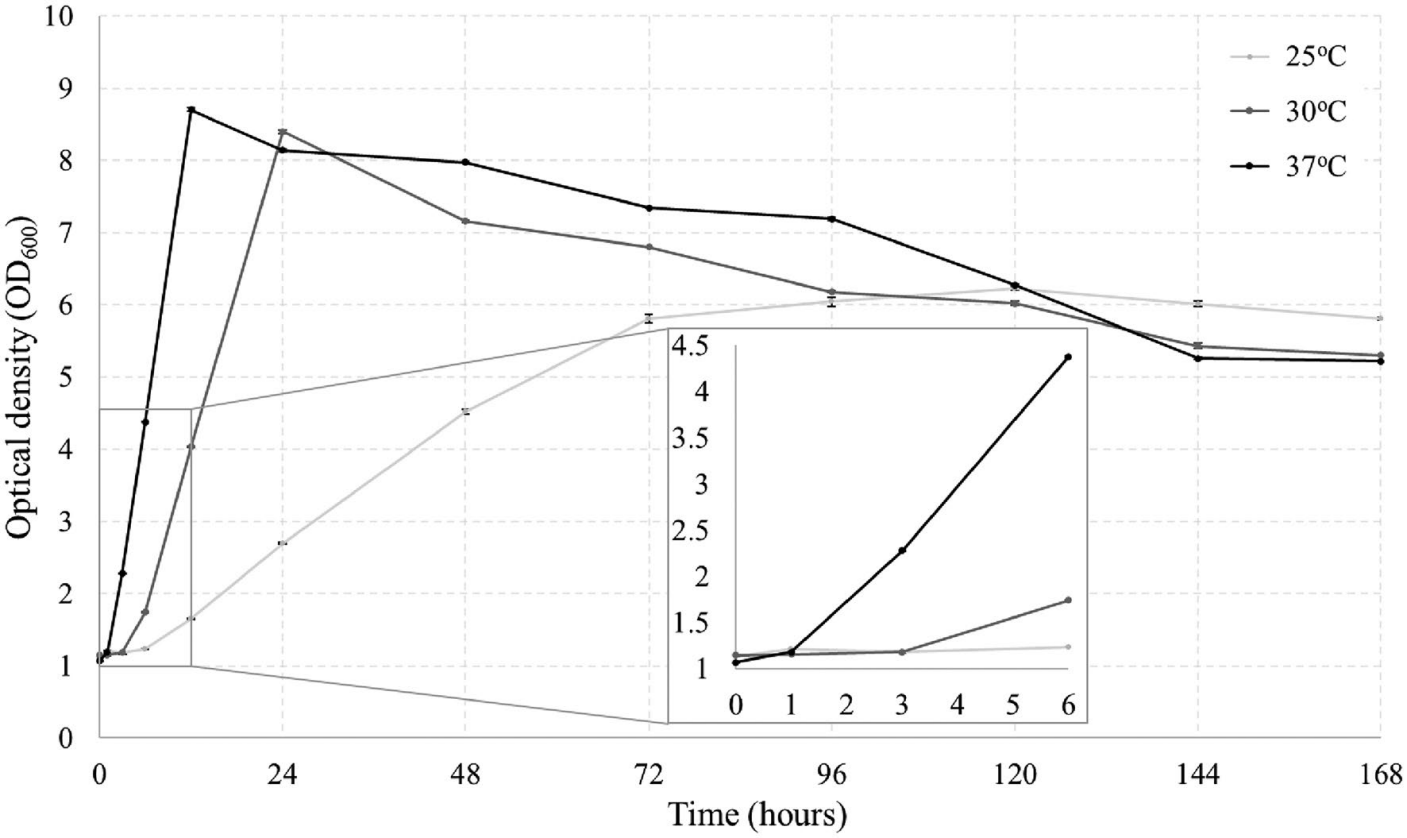
Growth of NBRC 14511 at different cultivation temperatures.

The growth curve, a representation of the live cell count in the bacterial population over time, was monitored using optical density (OD_600_) value (Fig. 6). Typically, bacterial growth curves are categorized into four distinct phases, namely, Lag Phase, Exponential Phase, Stationary Phase, and Death Phase, each delineating specific stages of bacterial growth dynamics^52^.

Observations revealed that the Lag Phase, characterized by cellular activity without noticeable growth, varied in duration across different temperatures. Specifically, the Lag Phase was shortest at 37 °C, lasting only 1 h, whereas at 30 °C and 25 °C, durations of 3 and 6 h were observed, respectively. Following the Lag Phase, bacteria transitioned into the Exponential Phase, during which cell division and doubling occurred rapidly, leading to a significant increase in cell concentration across all temperature conditions. The peak of Exponential Phase was observed at 12 h for 37 °C, 24 h for 30 °C, and 72 h for 25 °C, indicating temperature-dependent growth kinetics. The delineation of the Stationary Phase, where population growth begins to decline due to nutrient depletion and waste accumulation, varied with temperature. At 37 °C and 30 °C, Stationary Phase was not clearly discernible, as bacteria swiftly entered the Death Phase, marked by a gradual decrease in cell concentration over time. Conversely, at 25 °C, Stationary Phase was evident from 72 to 144 h, followed by entry into the Death Phase.

Furthermore, analysis of population curves and growth kinetics highlighted the strain NBRC 14511’s accelerated growth rate over time, particularly evident at higher temperatures. Notably, LAB strains exhibited optimal growth at 37 °C, with slower growth observed at lower temperatures, consistent with previously repoted growth optima for *Limosilactobacillus species*, which typically thrive between 30 and 40 °C^53,54^.

**Figure 7:**
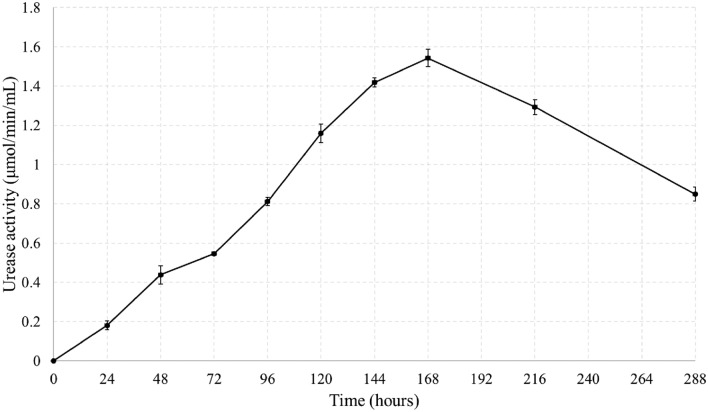
Stability of NBRC 14511 with the time at 37 °C.

Figure 7 illustrates the time stability of urease activity at 37 °C. The urease activity of the strain was tested daily until day 7, and additional tests were performed on days 9 and 12 to determine the peak. The results show that the highest urease activity is observed on day 7, indicating that a 7-day incubation period is optimal for precipitation and solidification experiments to achieve optimal efficiency.

#### Effect of pH

**Figure 8:**
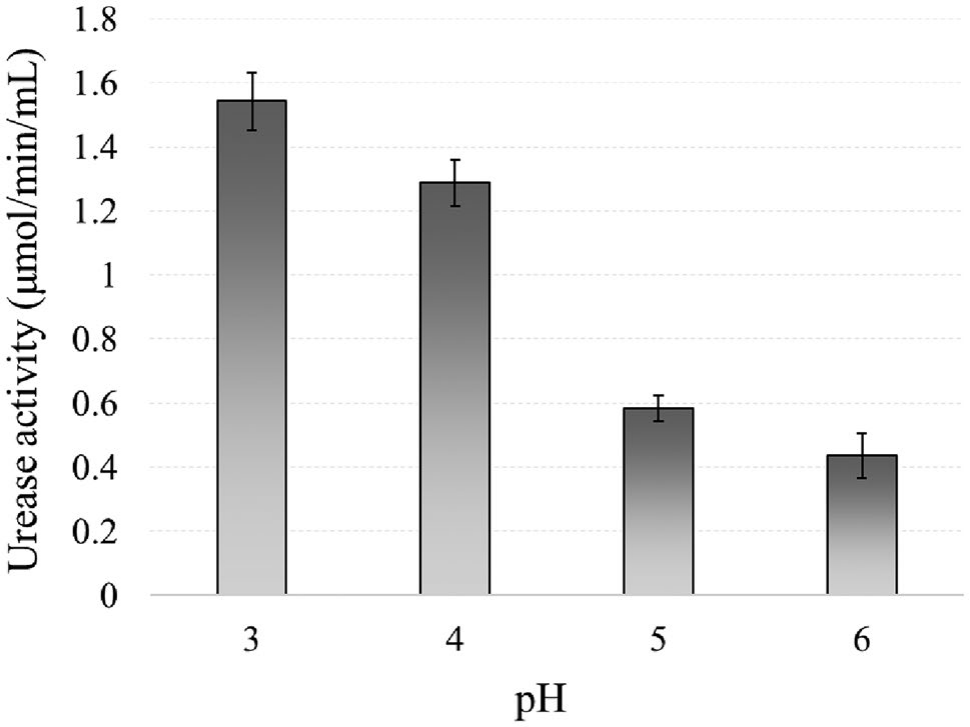
Effect of pH condition of reaction medium on NBRC 14511 performance.

After assessing the temperature, the next important factor to consider is the performance of the bacterium at different pH levels. The urease activity was evaluated at buffer pH levels 3, 4, 5, and 6, and the results are illustrated in Fig. 8. For successful precipitation of CPCs, the bacterium must be active at pH 3 and able to raise the pH of the solution to the level required for mineral precipitation. Given that FBS maintains a pH of 3, it serves as an ideal medium for the selected bacterial strain. The results of this experiment are in agreement with previous studies indicating that the optimal pH for *Lactobacillus* urease in vitro was determined to be pH 3–4^45,55^.

### Precipitation test

#### Observation during the treatment

**Figure 9:**
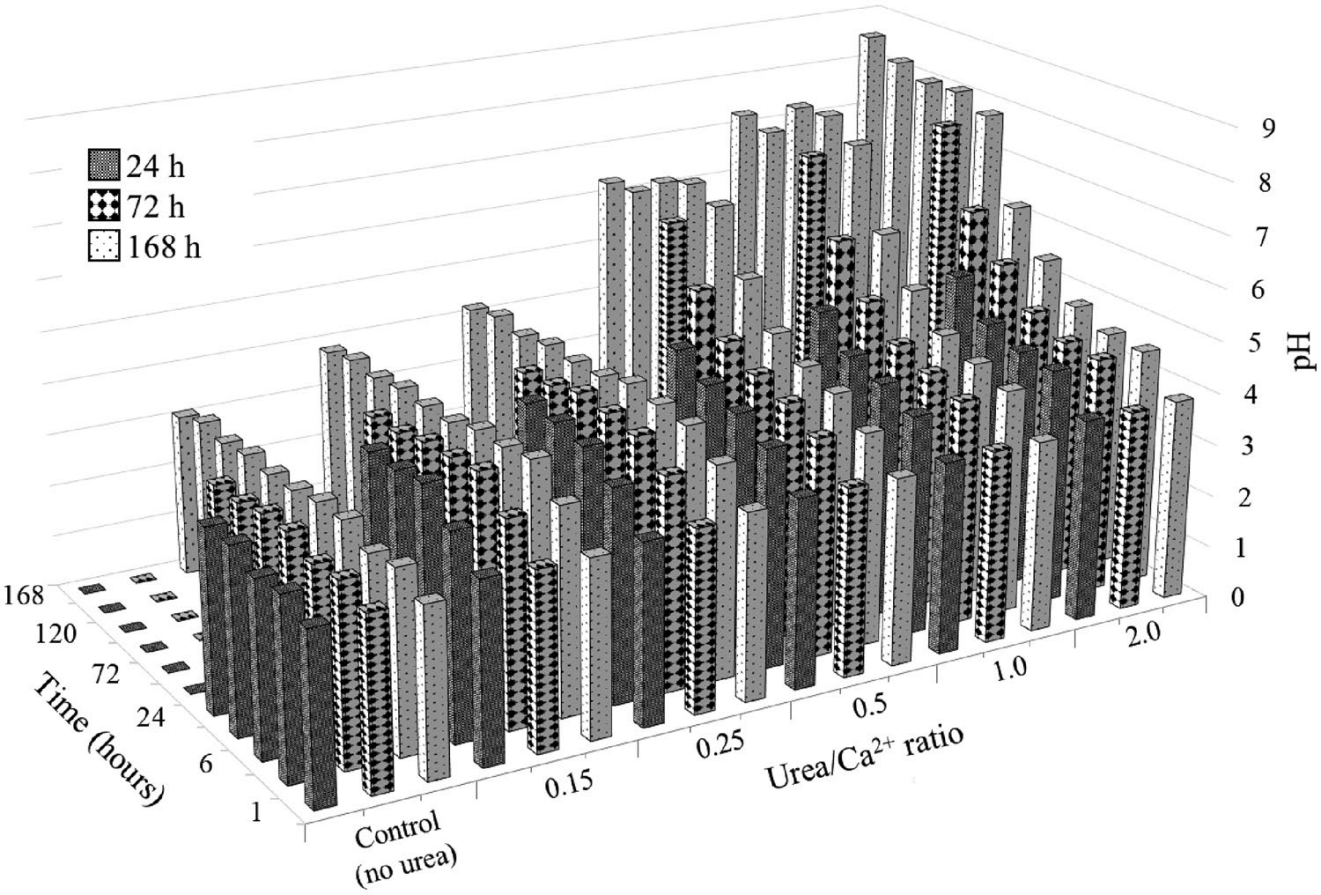
pH changes during precipitation test.

Important parameters in the deposition of CPCs are pH changes over time and Ca^2+^ concentration^56^. Given the significant influence of the pH of the medium on the deposition of CPCs, monitoring this parameter facilitates the evaluation of the effectiveness of formulation support and the course of the reaction. At the same time, the Ca^2+^ concentration measurement allows for a quick assessment of the deposition rate. Figure 9 illustrates the change in pH during deposition.

At low values of Urea/Ca^2+^ (0.15, 0.25), the pH level remains at the level of 4–4.5, thus minimizing the effect on the solidification test in the future. However, with an increase in the Urea/Ca^2+^ ratio, the pH increases noticeably, gradually moving from pH 3 to pH 8–9, thereby opening the way for further deposition of CPCs. A sample with a Urea/Ca^2+^ ratio of 2.0 demonstrates the most rapid increase in pH, reaching 5.45 after 24 h, 7.78 on day 3, and 8.54 on day 7.

Samples with a Urea/Ca^2+^ ratio of 0.5–2.0 show a similar tendency to rise in pH with a slight decrease after day 3, which may be explained by the complete dissolution of the DCPD, supplying the necessary ions to create a supersaturation in the solution necessary for HAp growth^57^. It is noteworthy that in the sample with a Urea/Ca^2+^ ratio of 0.5, a continuous decrease in pH was observed starting from day 4, indicating a constant conversion of DCPD to HAp. In contrast, at ratios of 1.0 and 2.0, this conversion occurs on days 6 and 5, respectively, resulting in a later increase in pH. The time of conversion of DCPD to HAp, indicated by pH reduction, decreases with increasing Urea/Ca^2+^ ratio, being the shortest (1 day) at 2.0 and the longest (3 days and beyond) at 0.5.

**Figure 10:**
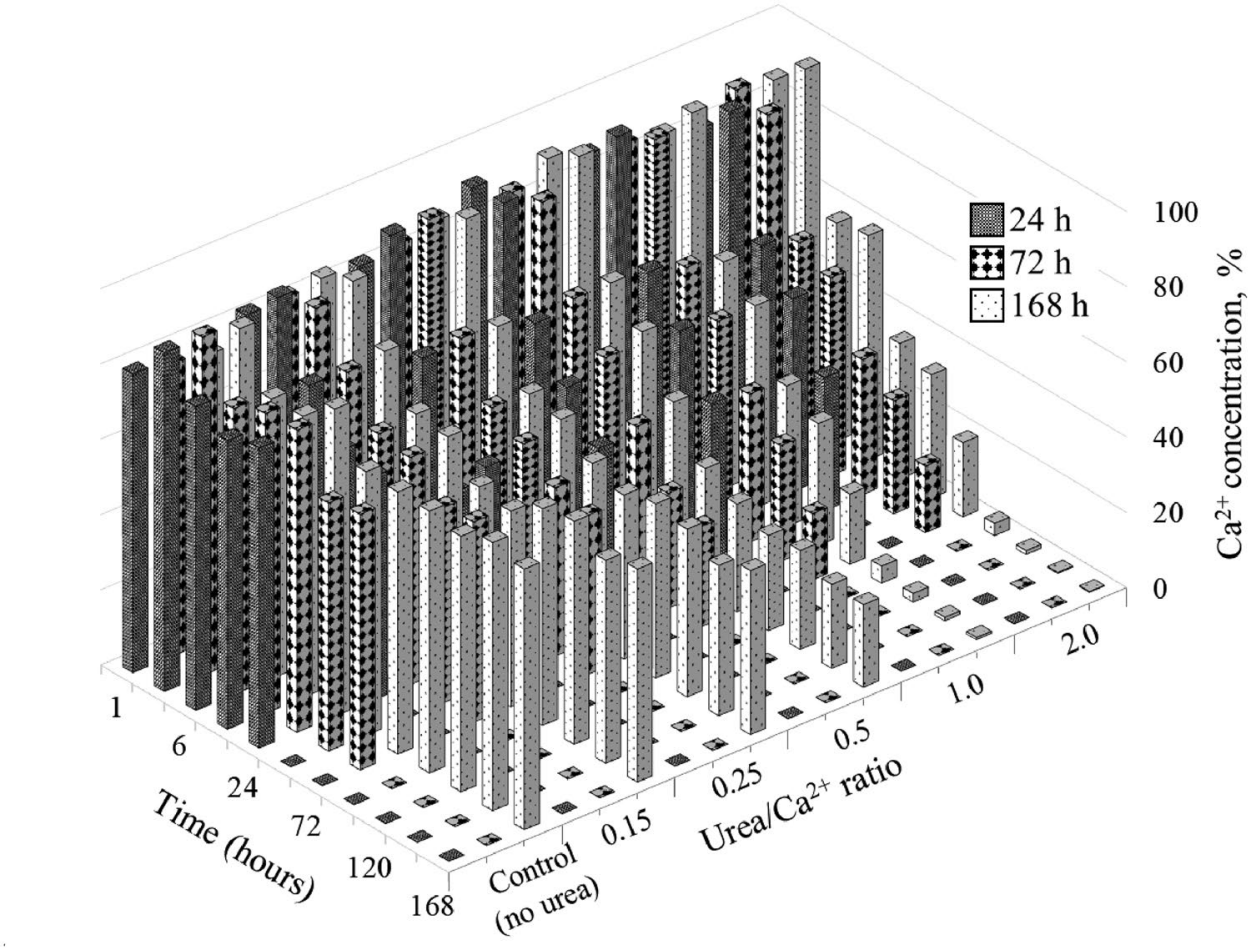
Ca^2+^ change during precipitation test.

Figure 10 shows the results of the Ca^2+^ concentration measurements during the precipitation experiment, which show a significant inverse relationship between the Ca^2+^ concentration and the pH of the system. As the Urea/Ca^2+^ ratio and pH increase, there is a noticeable and sharp decrease in the Ca^2+^ concentration, eventually reaching 0% in samples 1.0 and 2.0 on day 7.

During the first 24 h, all samples except the control exhibit a decrease in Ca^2+^ concentration, indicating the precipitation of DCPD and subsequent consumption of calcium and phosphate ions. After DCPD precipitation, at Urea/Ca^2+^ ratios of 0.15 and 0.25, Ca^2+^ consumption is less expressed compared to ratios from 0.5 to 2.0. Therefore, the pH of the medium, as shown in Fig. 9, might not be sufficiently favorable for the conversion of DCPD to HAp.

Nevertheless, a noticeable conversion occurs with different intensities for the ratios of 0.5, 1.0 and 2.0, indicating that a higher Urea/Ca^2+^ ratio correlates with a higher conversion rate. It is noteworthy that the observation on day 7 shows a 0% Ca^2+^ concentration for ratios of 1.0 and 2.0, possibly indicating that the conversion reaction is complete. Conversely, the ratio of 0.5 shows a 22% concentration with a decreasing trend, indicating an ongoing transformation. This discrepancy highlights the different reaction kinetics, with higher Urea/Ca^2+^ ratios indicating a faster and potentially more complete transformation reaction.

**Figure 11:**
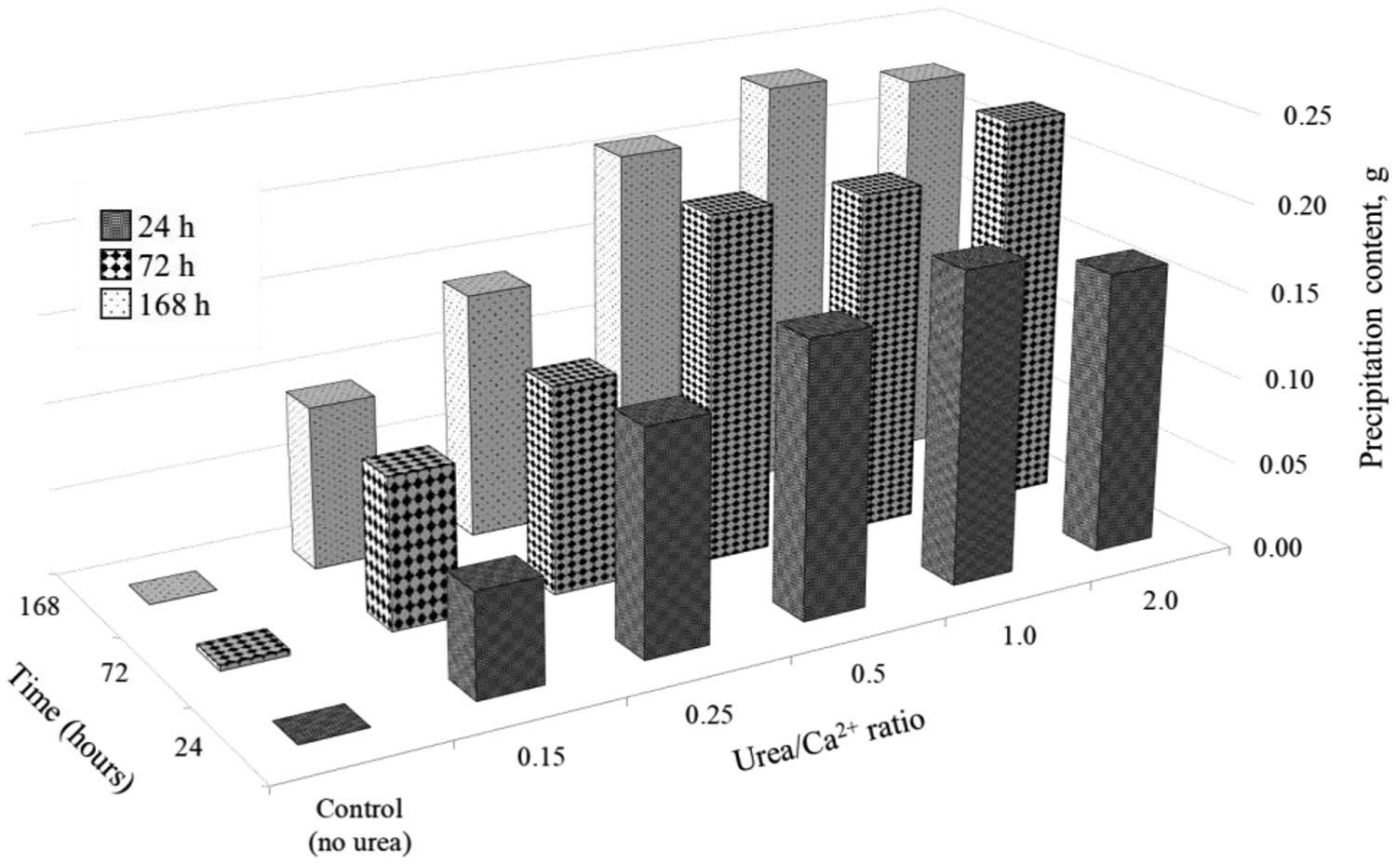
Precipitation content of samples of precipitation test.

The results of the precipitation test shown in Fig. 11 are consistent with the observed pH and Ca^2+^ utilization trends in the system, confirming the occurrence of CPCs precipitate. It should be noted that the ratios between 0.5 and 2.0 exhibit the highest amount of precipitation, making them particularly suitable for application in solidification tests.

#### XRD analysis of precipitation

**Figure 12:**
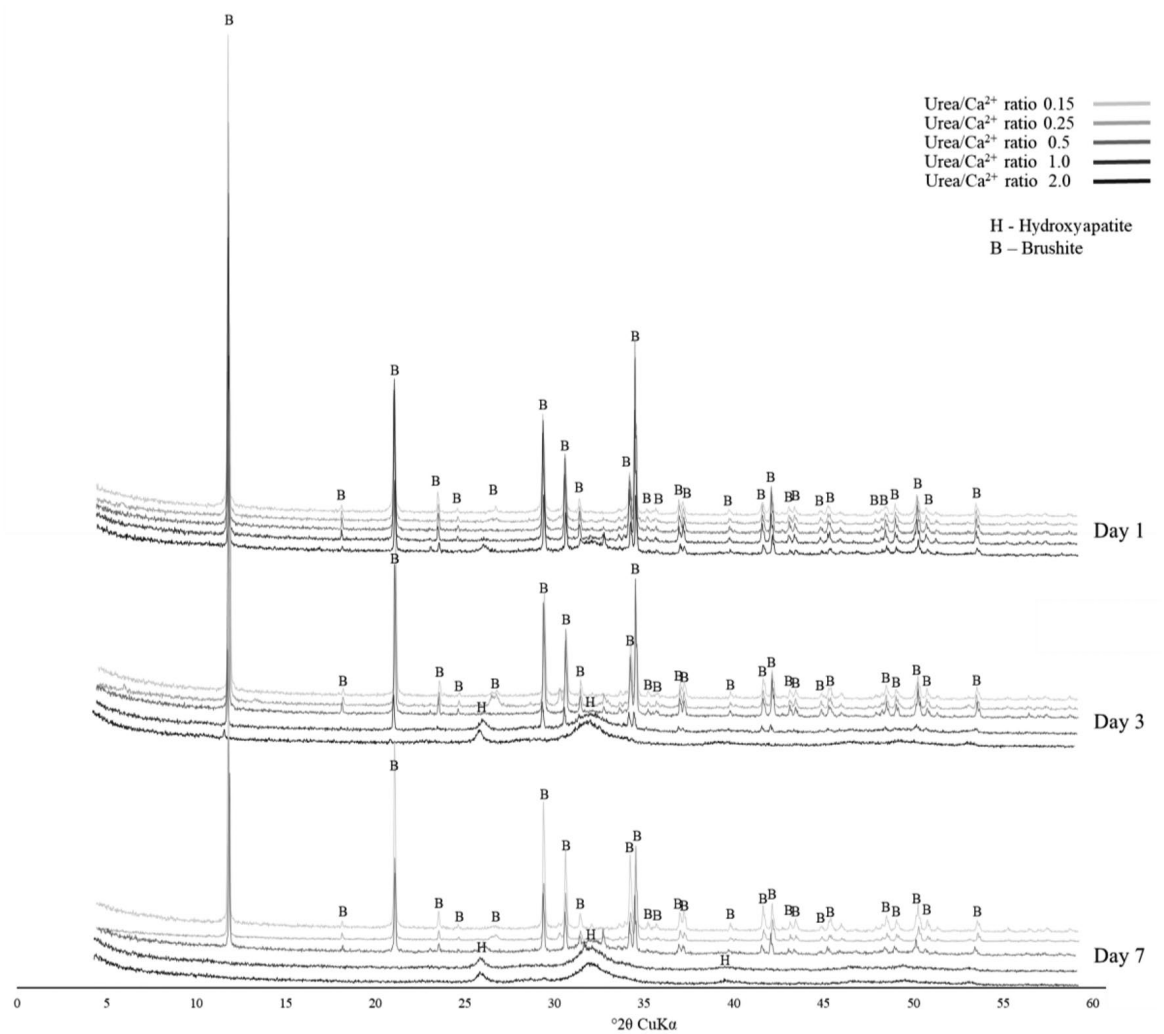
XRD analysis of precipitation test samples.

The XRD analysis of the deposits from the precipitation experiment reveals dynamic changes in mineral composition over time and in response to varying pH conditions, as shown in Fig. 12. The progression from brushite (mineral name of DCPD) to HAp is evident in shifting peaks, with a decrease in DCPD peaks at 11.7° and 20.9° corresponding to an increase in HAp peaks at 25.8° and 32.2°, which is consistent with previous publications^57,58^. This transformation is observed throughout the experiment from day 1 to day 7. At a Urea/Ca^2+^ ratio of 2.0, both DCPD and HAp peaks were observed on day 1, indicating the initiation of transformation within 24 h. On day 3, at a ratio of 2.0, the sample is almost completely recrystallized into HAp, while the sample with a ratio of 1.0 still contains both minerals, with more distinct HAp peaks. On day 7, both samples with a ratio of 1.0 and 2.0 completely change to HAp, eliminating the peaks of DCPD. Meanwhile, the sample with a ratio of 0.5 begins crystallization from DCPD to HAp, indicating a continuous transformation with increasing pH.

The precipitation sequence of CPCs is complexly regulated by thermodynamic solubility and kinetic factors. Under acidic conditions (pH 2–6), DCPD remains stable, serving as a precursor to HAp in alkaline environments^58–61^. However, some studies suggests that HAp becomes the most stable phase above pH 4, contrary to the traditional understanding^62,63^. This discrepancy emphasizes the nuanced interplay of pH conditions in determining the stability of different CPCs phases.

The aqueous transformation of DCPD into HAp reveals interesting dynamics. It is known that storing DCPD in aqueous solution causes its transformation into thermodynamically more stable phases and occurs most rapidly at around pH 7.5 to 8.0 at 40 °C^59,64^. The decrease in the time required for the conversion at higher temperatures is consistent with the nucleation and growth process, indicating the thermally activated nature of the reaction^58^. Additionally, the close relationship between DCPD and HAp indicates the potential for intergrowth, with HAp remaining the most stable phase after formation^63^.

While stoichiometric HAp traditionally adheres to an ideal Ca/P ratio of 1.67, the range for nonstoichiometric HAp remains uncertain. The position of the solubility isotherm for HAp strongly depends on the temperature of the solution and the Ca/P ratio. Though commonly accepted as 1.5, even lower values of 1.33 have been reported^65^. These nuances underscore the complexity of the transformation process and the sensitivity of the resulting phase to solution conditions.

DCPD precipitates from a liquid by creating supersaturation conditions due to higher concentrations of calcium and phosphate ions (Eq. 5)^56^.

5 $${\text{Ca}}^{2 + } + {\text{HPO}}_{4}^{2 - } + 2{\text{H}}_{2} {\text{O}} \to {\text{CaHPO}}_{4} \cdot 2{\text{H}}_{2} {\text{O}}$$

In an aqueous medium, the phase transformation from DCPD to HAp occurs through sequential dissolution and precipitation reactions, leading to the general reaction shown below (Eq. 6)^57^. Toward the end of the reactions, transformation rates decrease due to a diminishing HAp growth rate with decreasing supersaturation and size-dependent suppression of DCPD dissolution. This observation highlights the nuances of the reaction kinetics and the factors influencing the transformation rates throughout the experimental timeline.

6 $$10{\text{CaHPO}}_{4} \cdot 2{\text{H}}_{2} {\text{O}} + 8{\text{OH}}^{ - } \to {\text{Ca}}_{10} \left( {{\text{PO}}_{4} } \right)_{6} \left( {{\text{OH}}} \right)_{2} + {\text{4HPO}}_{4}^{2 - } + 26{\text{H}}_{2} {\text{O}}$$

#### SEM, SEM–EDS analysis of precipitation

**Figure 13:**
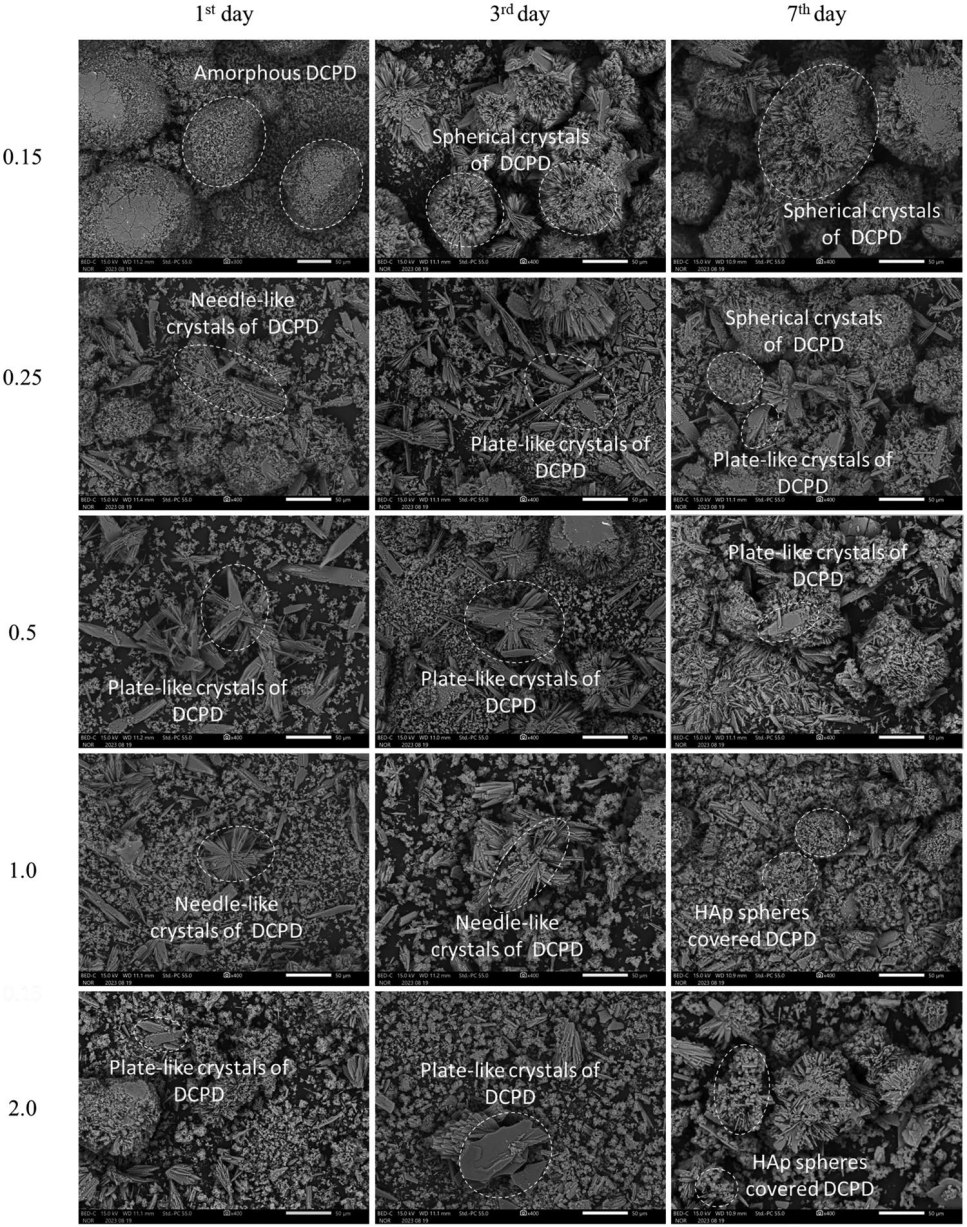
SEM images of precipitation test samples: horizontal axis—treatment duration (days), vertical axis—Urea/Ca^2+^ ratio within samples.

To investigate the microstructural changes during the deposition of CPCs, in particular DCPD and HAp, SEM and SEM–EDS analyzes were performed, the results of which are presented in Fig. 13 and Figs. 1A–3A in the "Supplementary Materials" section, respectively.

SEM images for a Urea/Ca^2+^ ratio of 0.15 show that after 24 h, undefined shape conglomerates of amorphous DCPD formation occurred. Over the following days, layered crystals developed on these neoplasms, creating a spherical structure, which is probably due to the stable pH and Ca^2+^ concentration in the solution until day 7. In the case of a ratio of 0.25, similar structures were observed with the deposition of layered DCPD crystals within 24 h, which may have been influenced by variations in growth intensity with pH.

At a relatively low pH (around 5), plate-like DCPD crystals typically formed, while at a higher pH (around 6.5), needle-like crystals precipitated^64^. At 0.5–2.0, the needle-like neoplasms appeared alongside the plate-like ones, especially noticeably at 0.5 on day 7, confirming the previous statement.

The hydrolysis of DCPD in an alkaline solution releases calcium and phosphorus ions, and the DCPD lattices serve as precursors and templates for the oriented nucleation of HAp. This process results in a highly oriented hierarchical structure during the transformation, covering the crystal surface and growing from the surface to the core^58,66^*.* Moreover, HAp obtained from DCPD shows dense aggregates of irregular thin microcrystals^67^.

While numerous studies have explored various morphologies of HAp crystals, including nano-flakes, rods, needles, flower-like spheres, plates, and nanoparticles^68^, the typical form of hydroxyapatite is nano-needle-like morphology^69,70^. However, in this study, hydroxyapatite precipitated as nanoparticle agglomerates with round morphology, which was enhanced in cases 1.0 and 2.0 on day 7. This round morphology, previously reported in studies^71–73^, is evident in images for 1.0 and 2.0, illustrating that hydroxyapatite has completely covered the brushite crystals by day 7 and is growing inside the core. Consequently, no peaks for DCPD were found in the XRD results for these samples.

SEM–EDS images supported these findings, confirming the formation of CPCs in all samples (Figs. 1A–3A).

### Sand solidification test

#### Observation during the treatment

**Figure 14:**
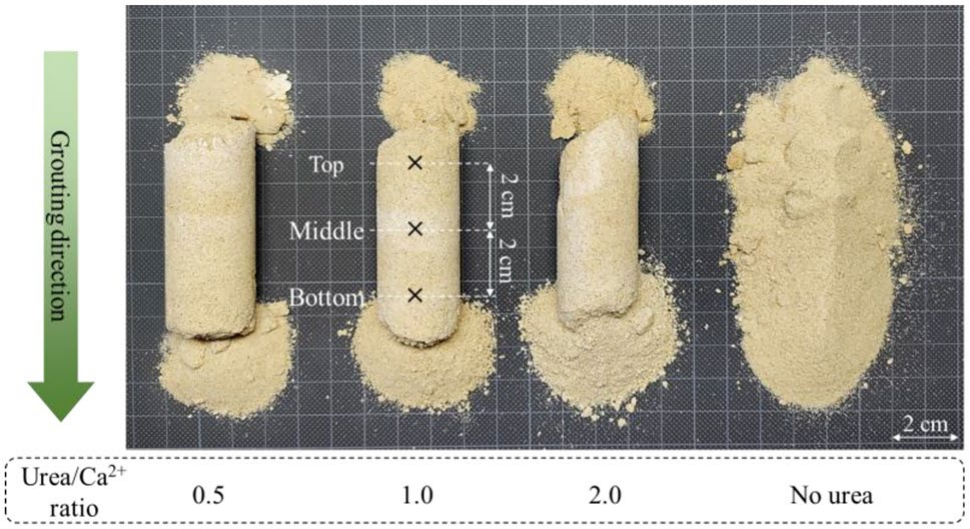
Photo of sand solidification test cases.

Selected Urea/Ca^2+^ ratios from the precipitation test were used in the solidification experiment, and the results are shown in Fig. 14. This figure provides a visual representation of the appearance of the specimens after curing, indicating the locations selected for UCS, precipitation content tests, and SEM analyses. Notably, observations made after a 14-day period revealed inadequate compaction of both the bottom and top levels within each sample, compromising their cohesion. It is noteworthy that the sample with a Urea/Ca^2+^ ratio of 0.5 had a more stable cylindrical shape compared to the others, while the control sample did not show a significant increase in strength over time. As the Urea/Ca^2+^ ratio increased, and consequently, the urea concentration within the samples rose, sand particles exhibited reduced cohesion at the lower portion of the samples, resulting in a greater proportion of loose sand, thereby diminishing overall strength contributions.

**Figure 15:**
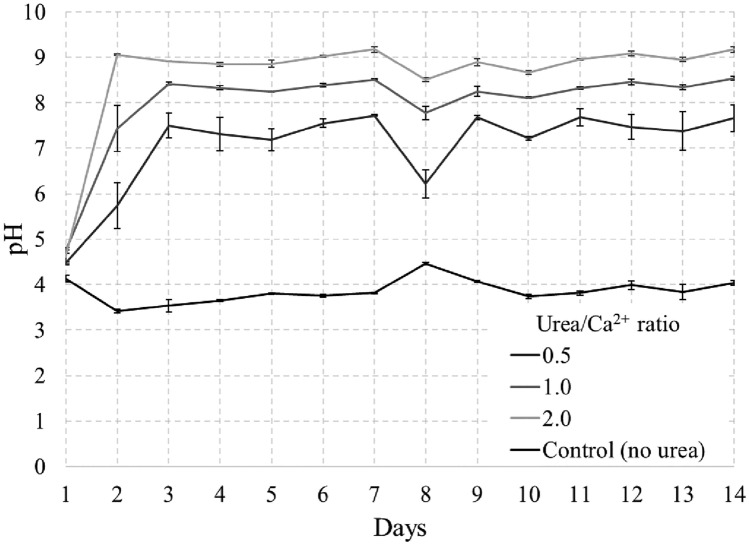
pH changing during solidification test.

Figure 15 demonstrates the pH changes over time in the samples, showing a sharp initial increase during the first 1–2 days, which is attributed to the hydrolysis of urea by the bacterium (Eq. 4). Starting from day 3, the pH level remained relatively constant. In particular, the pH reached a maximum value of 7.72 for a ratio of 0.5, 8.53 for a ratio of 1.0, and 9.17 for a ratio of 2.0. However, a noticeable decline in pH on day 8 could be attributed to the second bacterial injection the day before, which unbalanced the system. The pH change observed during the solidification test indicates that the bacterium exhibits a comparable behavior within the sand matrix as in a medium environment. During the precipitation test, the pH attained maximum values of 7.07 for a ratio of 0.5, 7.99 for a ratio of 1.0, and 8.54 for a ratio of 2.0 (Fig. 9). Presumably, the sand particles and the voids between them provide a limited space for the bacteria, encouraging them to hydrolyze urea more intensively, which leads to higher pH levels.

**Figure 16:**
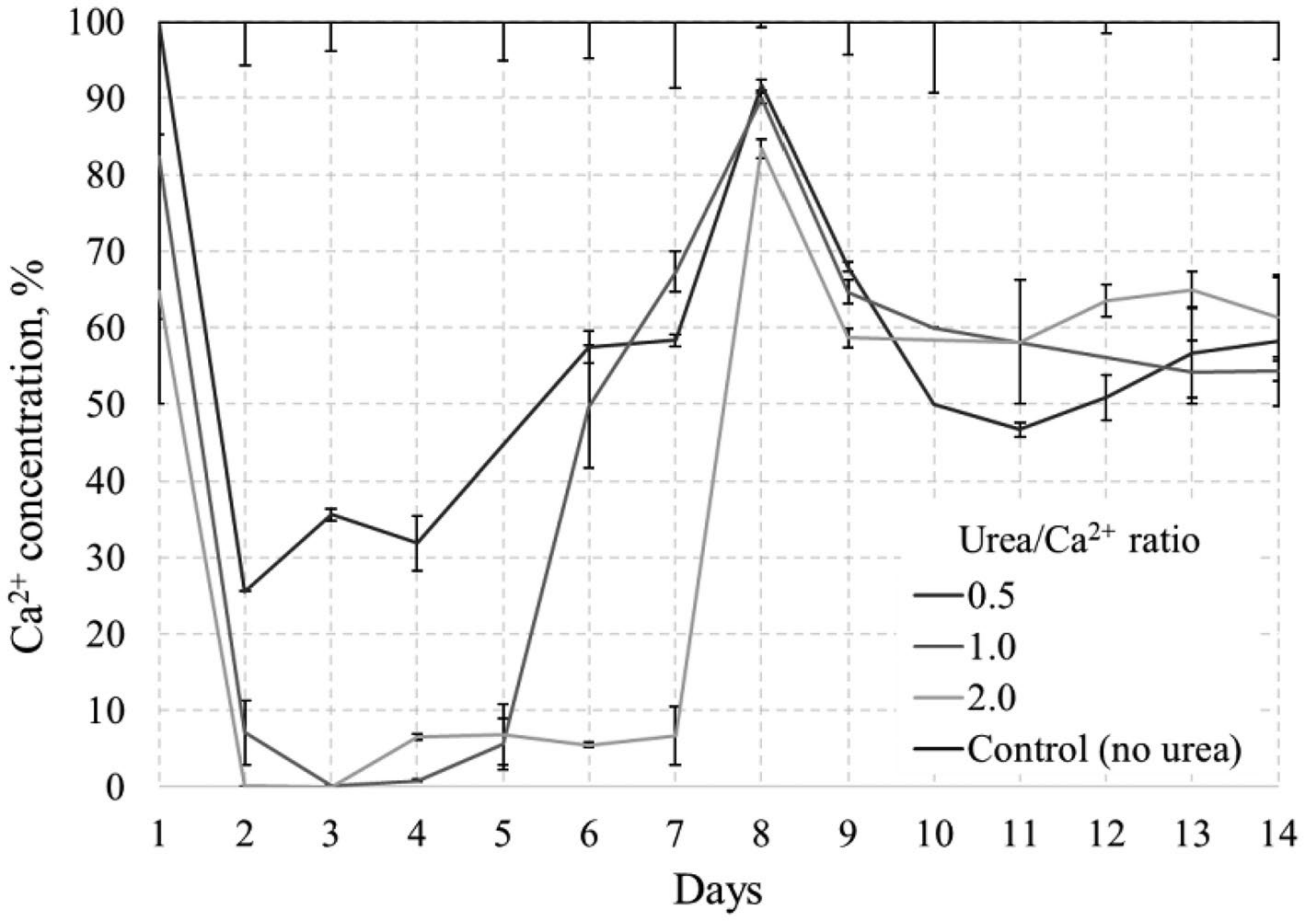
Ca^2+^ concentration changing during solidification test.

The time progression of Ca^2+^ utilization in the system, indicating the precipitation of CPCs, is shown in Fig. 16. The scale is calibrated to indicate that 100% corresponds with zero Ca^2+^ reduction or precipitation in the control sample. It is noteworthy that the first day is the most effective for all samples, when the Ca^2+^ concentration decreases from 75%–100% to 0%. The smallest decrease is observed in the case of a ratio of 0.5, while the most significant reduction is observed in the case of a ratio of 2.0. Figure 16 shows a consistent pattern with the pH results, where on day 8 all samples show an increased Ca^2+^ content due to the destabilization of the system after the addition of the bacterial medium. These findings may indicate accelerated hydrolysis reactions and heightened consumption of Ca^2+^ at a faster rate, supporting previous pH findings. After 48 h, Ca ion concentrations were 25% for a ratio of 0.5, 7% for a ratio of 1.0, and 0% for a ratio of 2.0, compared to the precipitation test where they were 33%, 32%, and 32%, respectively, for equivalent urea concentrations (Fig. 10).

**Figure 17:**
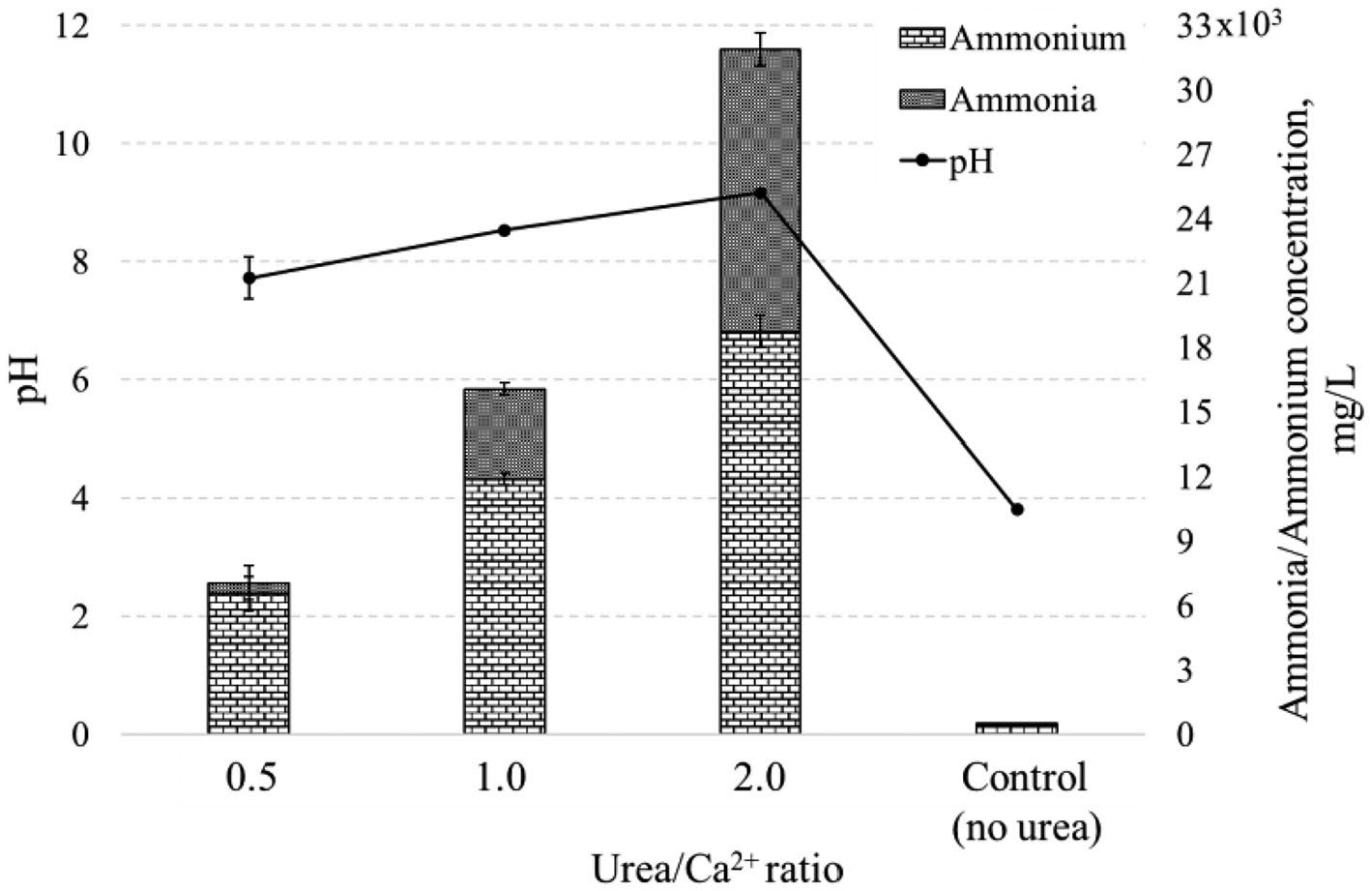
Ammona and ammonium concentration changing during solidification test.

The environmental impact of soil compaction methods is crucial, especially regarding the release of harmful substances into the soil environment. According to Japan's Unified National Wastewater Standards, the acceptable limit for ammonia in the soil environment is 100 mg/l^74^. Figure 17 illustrates the maximum ammonia concentrations released during the experiment. The ammonium content was measured and converted to ammonia, considering the experimental conditions—temperature of 37 °C and pH of the solution^75^. The results of the study show a tendency to increase the ammonia concentration with an increase in the Urea/Ca^2+^ ratio: 503 mg/l for a ratio of 0.5, 4159 mg/l for a ratio of 1.0, and 13,140 mg/l for a ratio of 2.0. It should be noted that the 0.5 ratio is close to the limit value, making it potentially suitable for use in open field applications in the future. Despite exceeding standard limits, these values are significantly lower than conventional MICP-induced ammonia emissions, often exceedingly at least 10,500 mg/l^76^. Application of MICPP in soil stabilization could involve ammonia utilization as fertilizer through careful planning and precautions^77^. Another strategy for controlling ammonia emissions during MICPP could involve reducing the urea content within the medium. This approach aims to regulate low pH levels, as there is a direct correlation between ammonia emissions and pH. Additionally, exploring areas such as bio-brick production using MICPP could offer a solution by managing ammonia outside the groundwater environment^78^.

#### UCS and precipitation content

**Figure 18:**
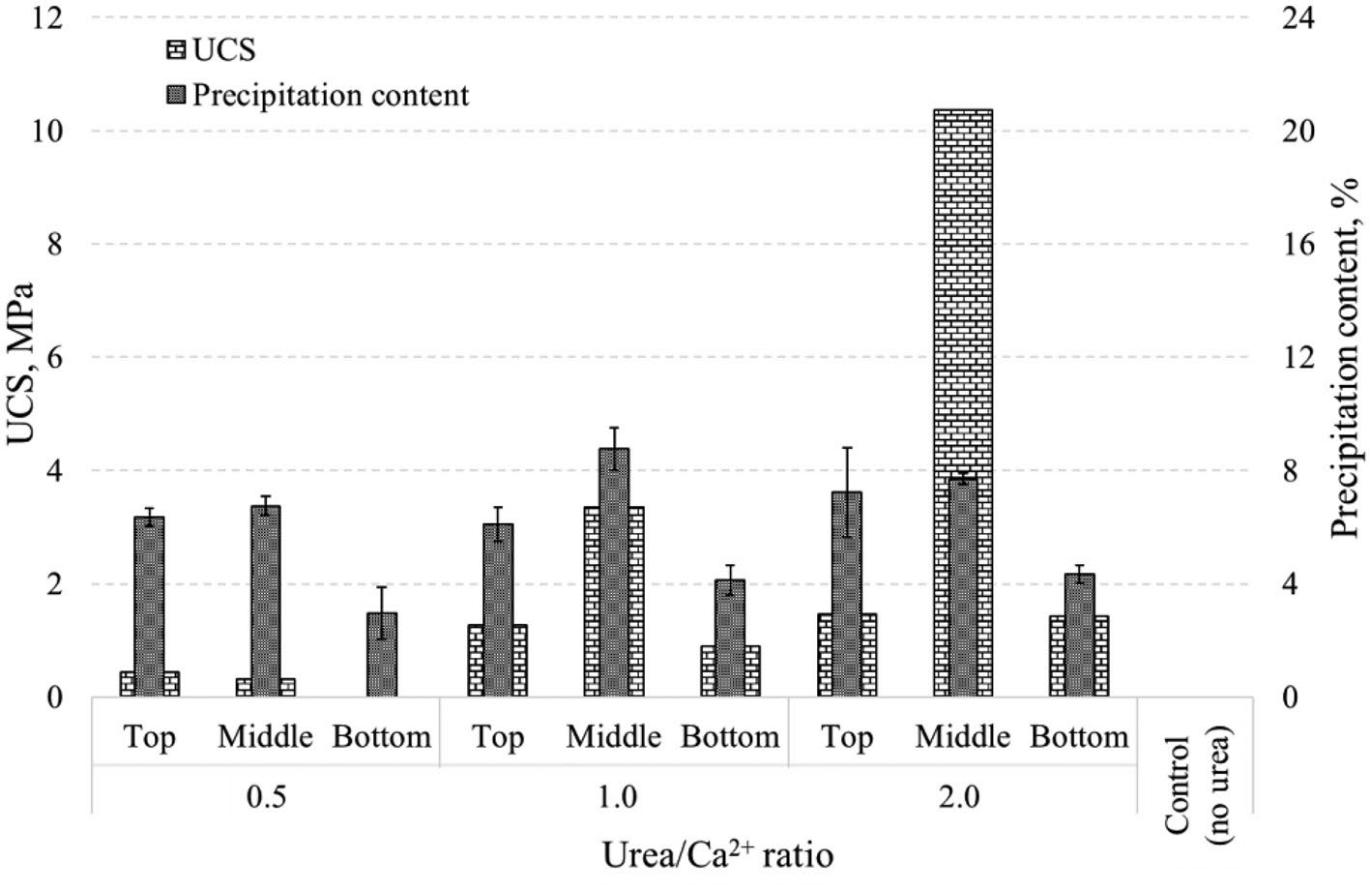
UCS and precipitation content results.

Figure 18 depicts the UCS and precipitation content results for all samples investigated in this study. Notably, sample with Urea/Ca^2+^ ratio of 2.0 exhibited a substantially higher strength of 10.35 MPa compared to sample 1.0, which registered a UCS of 3.34 MPa, despite both samples having the same precipitation content. This observation raises intriguing questions regarding the role of mineral composition in the precipitated CPCs. In previous studies using urease and FBS by the Enzyme Induced Calcium Phospate Precipitation (EICPP) method with Urea/Ca^2+^ ratios ranging from 0.5 to 2.0, the maximum UCS achieved was 6.05 MPa for a ratio of 2.0, 5.76 MPa for a ratio of 1.0 and 3.4 MPa for a ratio of 0.5^19^. In contrast, conventional MICP typically achieves results of 0.53–1.5 MPa for a Urea/Ca^2+^ ratio of 1.0 employing silica sand with d_50_-0.2–0.74 mm^79^. Utilizing China standard sand with a particle size distribution of d_10_-0.16 and d_50_-0.74, the MICP method achieved sand enhancement up to UCS of 1.5 MPa^80^. Otawa sand (d_10_-0.31, d_50_-0.45) and Mississippi sand (d_10_-0.2, d_50_-0.33) exhibited UCS values of 1.36 MPa and 0.53 MPa, respectively^81^. Additionally, fine silica sand with a particle size distribution of d_10_-0.16 and d_50_-0.23 demonstrated a UCS of 1.1 MPa^82^. Quartz sand, characterized by a particle size distribution of d_10_-0.15 and d_90_-0.3, exhibited a UCS of 1.0 MPa^83^. Moreover, Ottawa sand (d_10_-0.28, d_50_-0.48) displayed a UCS of 1.2 MPa^84^.

Based on the current test results for a Urea/Ca^2+^ ratio of 1.0, it can be concluded that MICPP offers advantages over traditional MICP in the case of improving silica sand with a particle size distribution of d_10_-0.15 and d_50_-0.27. This demonstrates superior strength and efficiency in binding fine sand particles by CPCs.

#### XRD analysis of solidification samples

**Figure 19:**
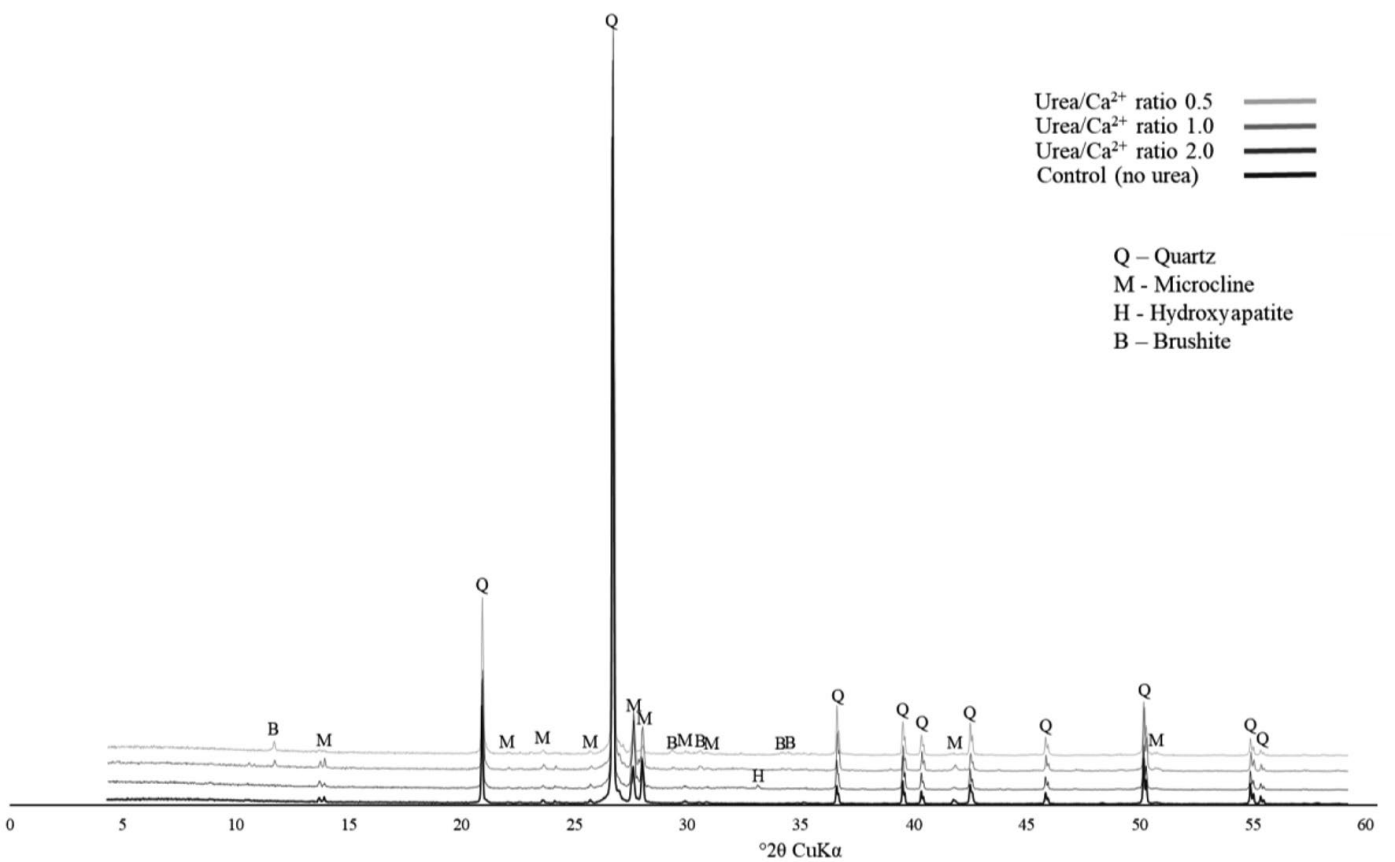
XRD of solidification test samples.

To further investigate the sand solidification and strengthening process using CPCs, XRD analysis of the samples was performed, and the results are shown in Fig. 19. Following the results of the precipitation experiment, the samples with lower Urea/Ca^2+^ ratios showed peaks exclusively for DCPD. In particular, the 0.5 sample showed clear peaks for quartz, microcline (a natural component of Toyoura sand), as well as peaks for DCPD. In contrast, only the sample with a concentration of 2.0 showed no peaks for DCPD and peaks for HAp. Although samples with Urea/Ca^2+^ ratios ranging from 0.5 to 2.0 displayed distinct peaks for HAp after 7 days of precipitation testing, only the sample with a ratio of 2.0 exhibited the absence of DCPD peaks and the presence of HAp peaks after the solidification test. Additionally, the sample with a Urea/Ca^2+^ ratio of 1.0 exhibited smaller peaks for DCPD, indicating the initiation of the conversion from DCPD to HAp. This suggests that high urea concentration conditions are more conducive to HAp formation, and the conversion from DCPD to HAp occurs over a longer duration in the sand environment compared to the stable medium environment.

#### SEM, SEM–EDS of solidification samples

**Figure 20:**
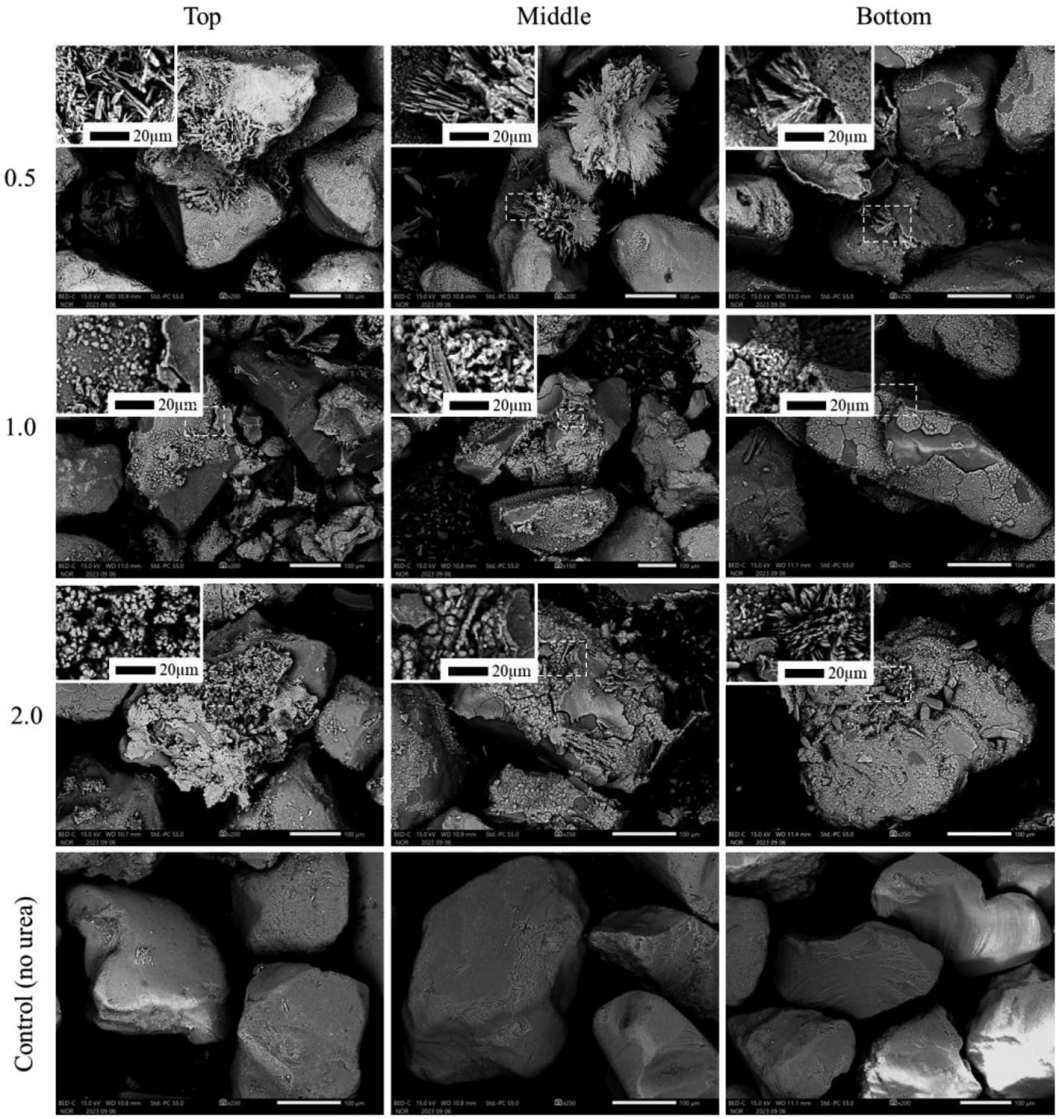
SEM of solidification test samples: horizontal axis—location of SEM image, vertical axis—Urea/Ca^2+^ ratio within samples.

The SEM results are consistent with the XRD results, revealing a needle-like morphology of the DCPD crystals at all layers in the 0.5 sample (Fig. 20). This shape of the DCPD crystals is typical of DCPD precipitation at pH levels above 6.5^64^. Although the XRD results for sample 1.0 did not show peaks for HAp, SEM images indicate the initial growth of spherical HAp crystals on the surface of the DCPD crystals, resulting in the intergrowth of needle-like DCPD crystals and HAp spheres. In the case of ratio 2.0, the top and middle levels have undergone a complete transformation from DCPD to HAp, while the bottom level still contains both DCPD and HAp crystals. This observation explains the reason for the higher UCS values for the middle part of this sample compared to other samples with similar sediment content (Fig. 18).

To confirm the formation of CPCs, SEM–EDS analysis was performed. In Fig. 4A in the “Supplementary Materials” section, the images illustrate the distribution of Si, Ca, and P in the samples from the solidification experiment. The results show that calcium and phosphorus coexist in the identical areas, strongly supporting the existence of CPCs formations in the samples.

### Future potential of the MICPP

The economic viability of MICPP lies in its potential to utilize sustainable materials such as tuna fish bones, a byproduct of the tuna production industry. A comprehensive cost analysis was conducted to compare MICPP with conventional MICP, based on reagent prices in the Japanese market. The results, detailed in Table 1, indicate that for treating 1 m^3^ of silica sand over a 14-day period, MICPP incurs a total reagent cost of 3,066,961.5 JPY, whereas MICP amounts to 2,268,173.4 JPY. Initially, MICPP appears 1.35 times more costly than MICP; however, leveraging inexpensive sources of acid and alkali can bring down the cost to 0.8 times cheaper than MICP. The main cost factor in the application of MICCP, accounting for more than 60% of the costs, is related to the utilization of hydrochloric acid. By replacing it with natural acids, such as citric, acetic, lactic, malic, tartaric, amino acids, humic and sulfuric acids, significant cost savings can be achieved^85^. An alternative avenue for cost optimization involves shortening the sand treatment duration. Experimental findings in this paper revealed comparable precipitation levels on the 3rd and 7th day suggest the feasibility of reducing treatment duration and subsequently cutting costs. Moreover, cost reduction could be achieved by substituting artificial urea with animal urine. This optimization of the MICPP method not only aids in cost reduction but also contributes to mitigating environmental pollution caused by animal waste while offering a resource substitution solution^86–87^.

**Table 1:** Cost comparison between MICPP and MICP treatment of 1 m^3^ silica sand.

Reagents	MICPP				MICP^88^			
	Substance	Required amount (kg[L])	Unit price (JPY/kg[L])	Price (JPY)	Substance	Required amount (kg[L])	Unit price (JPY/kg[L])	Price (JPY)
Cementation solution	Urea	140.0	3640.0	509,600.0	Urea	140.0	3640.0	509,600.0
	Fish bone	466.7	0.0	0.0	CaCl_2_	166.5	6200.0	1,032,052.0
	HCl	625.0	2100.0	1,312,509.4	Nutrient Broth	9.0	29,000.0	259,840.0
	NaOH	28.9	3860.0	111,423.5				
Culture solution	MRS Broth	36.8	30,800.0	1,133,428.7	Yeast Extract	10.0	36,400.0	363,854.4
					(NH_4_)_2_SO_4_	5.0	3520.0	17,593.0
					Tris-Buffer	7.9	10,833.0	85,234.0
Estimation	Total material cost			3,066,961.5	Total material cost			2,268,173.4

Future research directions could delve into the durability of CPCs and their morphological transformations over time. In addition, attempts to control the Ca/P ratio in the medium to precisely control mineralization are worthy of attention. Furthermore, fiber integration stands out as a compelling strategy to enhance the strength of MICPP-treated sand or soil, thereby increasing the UCS and simultaneously reducing ammonia emissions.

Despite its initial cost, MICPP offers sustainability benefits by utilizing fish-processing waste, rendering it more environmentally friendly compared to conventional MICP biogrout^21^. Additionally, employing MICPP has the potential to reduce ammonia emissions by up to 95%, as illustrated in Fig. 17, paving the way for larger-scale soil solidification projects.

In terms of material strength, studies have recorded varying compressive strengths for MICP bio-bricks, ranging from 2.7^89^ to 9 MPa^90^. While MICPP bio-bricks have not been fabricated yet, current findings suggest the potential for even greater strength compared to conventional MICP method. Moreover, MICPP proves effective in crack remediation, with observed concrete or stone clogging within a short timeframe of just two days post-treatment^21^.

Addressing soil-related challenges, MICPP's requirement for an acidic environment makes it particularly suitable for treating acidic soils (pH < 5.5), which cover a significant portion of the world's ice-free land^91^. Furthermore, MICPP has shown promise in removing heavy metal contaminants from soils, such as lead in Pb–Zn tailings, showcasing its potential for environmental remediation^92^.

Regarding soil stabilization, the efficacy of MICPP is underscored by its ability to achieve a UCS value of 0.345 MPa or higher, a critical threshold for efficient soil stabilization^93^. Moreover, MICPP can mitigate soil liquefaction, as UCS values exceeding 0.1 MPa are indicative of its effectiveness in this regard^94^.

Beyond soil applications, MICPP could find utility in diverse areas such as slope stabilization, where studies on MICP have successfully enhanced slope cover conditions with average UCS ranging from 2 to 8 MPa^95^ making MICPP method with UCS results of 3.34 and 10.35MPa viable for application. Additionally, MICPP holds promise for addressing wind erosion, internal erosion control, and airborne dust control.

## Conclusions

This study presents a novel method, MICPP, to improve soil stability through the deposition of CPCs. The use of *Limosilactobacillus sp.*, in particular strain NBRC 14511, in combination with FBS allowed to precipitate DCPD and HAp, especially at 37°C and initial pH 3.

In the precipitation experiments, dynamic changes in pH and Ca^2+^ concentration were observed, which influenced the precipitation and transformation of DCPD into HAp. Subsequent XRD and SEM analyses confirmed this transformation, highlighting the clear microstructural characteristics of the formed HAp.

MICPP performed better than conventional MICP in sand solidification experiments with different Urea/Ca^2+^ ratios, resulting in higher UCS. Microstructural analysis revealed the mineral composition, showing that higher Urea/Ca^2+^ ratios resulted in the production of HAp, which contributed to the strength increase.

The environmental impact assessment, including ammonia concentration, showed values within acceptable limits for potential field application. In particular, the sample with the lower Urea/Ca^2+^ ratio (0.5) proved to be a viable option for soil consolidation in the field, although with certain limitations to control ammonia emissions. Samples with higher ratios (1.0 and 2.0) demonstrated potential applications in bio-brick production or soil compaction, where effective control of ammonium emissions is possible.

In summary, the study demonstrates that MICPP is a promising and sustainable method of soil stabilization that provides improved mechanical properties and reduced environmental impact compared to traditional approaches. The combination of LAB and calcium and phosphorus sources from fish bones is an environmentally friendly solution for soil engineering that eliminates the limitations of traditional stabilization methods and promotes sustainable and novel practices in geotechnical applications.

### Supplementary Information

Supplementary Figures.

## Data Availability

All data generated or analyzed during this study are included in this published article.
