# Supplementary material for: Ecofriendly solidification of sand using microbially induced calcium phosphate precipitation
Source: Sci Rep. 2024 May 30;14:12412. doi: 10.1038/s41598-024-63016-9 (PMC11139996; doi:10.1038/s41598-024-63016-9)
Supplement: Supplementary file 1 — Supplementary Figures. [file 41598_2024_63016_MOESM1_ESM.pdf]

Appendix A: Supplementary material

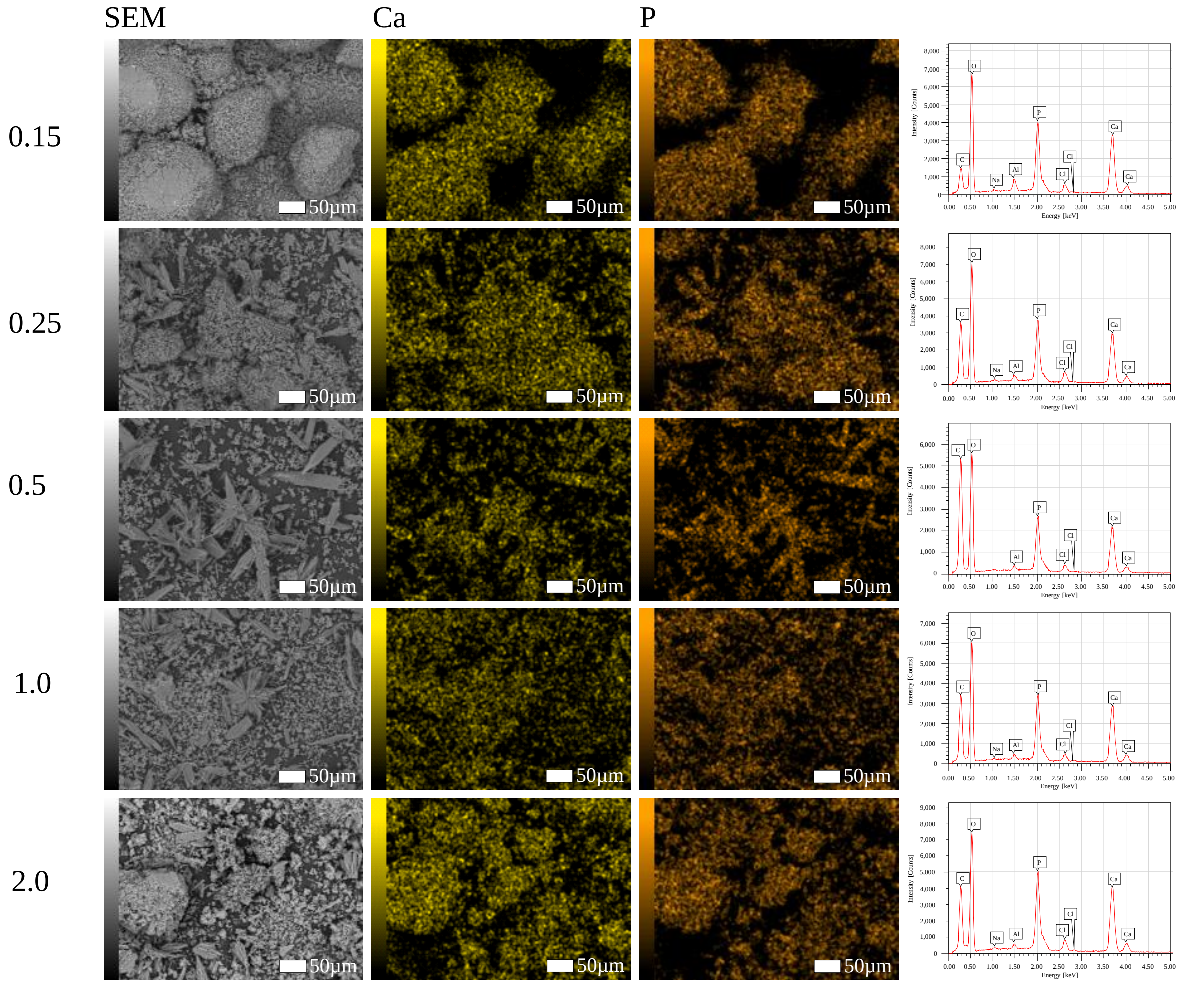

Fig. 1A. *The SEM-EDS analysis of precipitation samples on the 1st day: vertical axis – Urea/*Ca^{2+} *ratio within samples.*

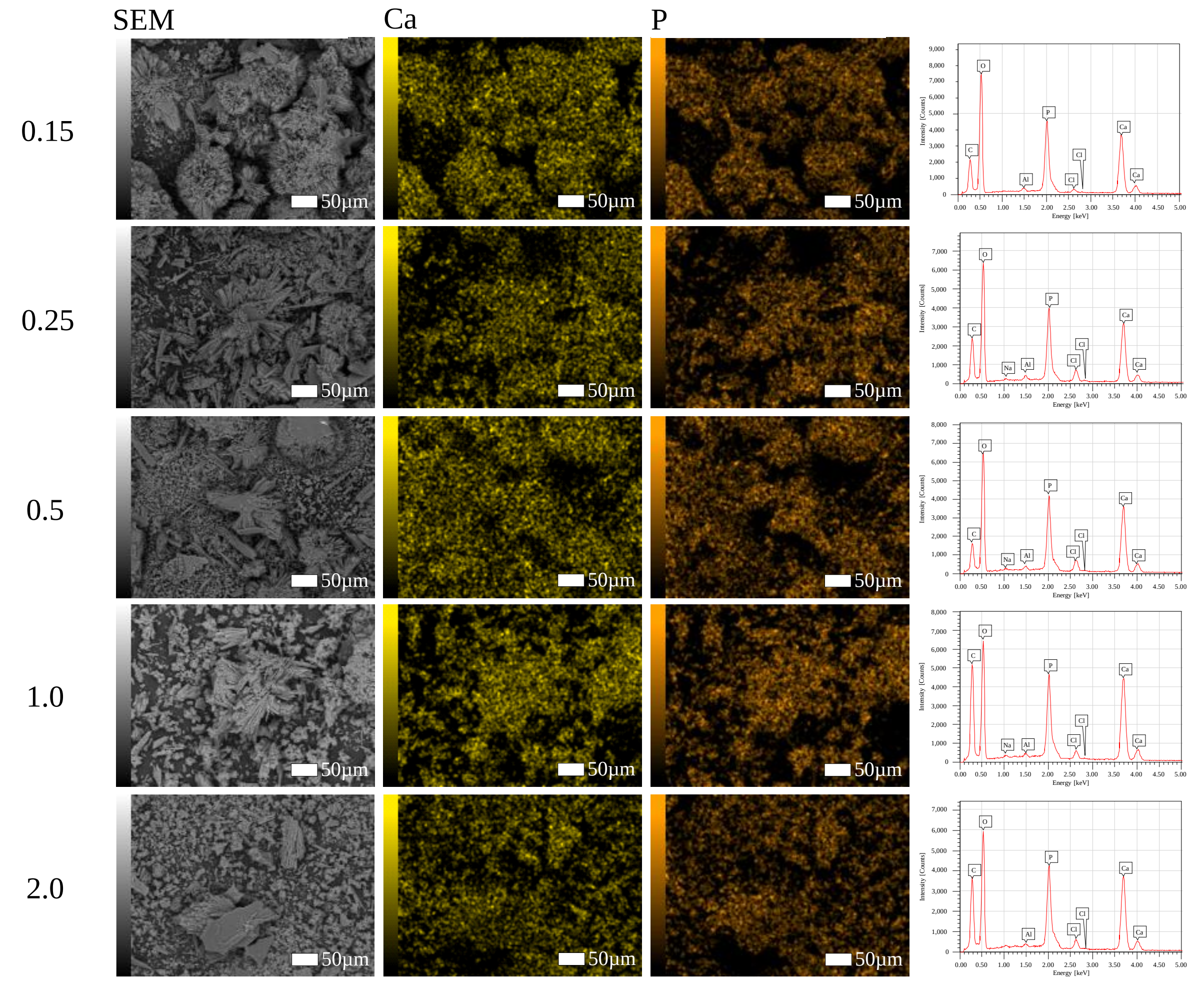

Fig. 2A. *The SEM-EDS analysis of precipitation samples on the 3rd day: vertical axis – Urea/*Ca^{2+} *ratio within samples.*

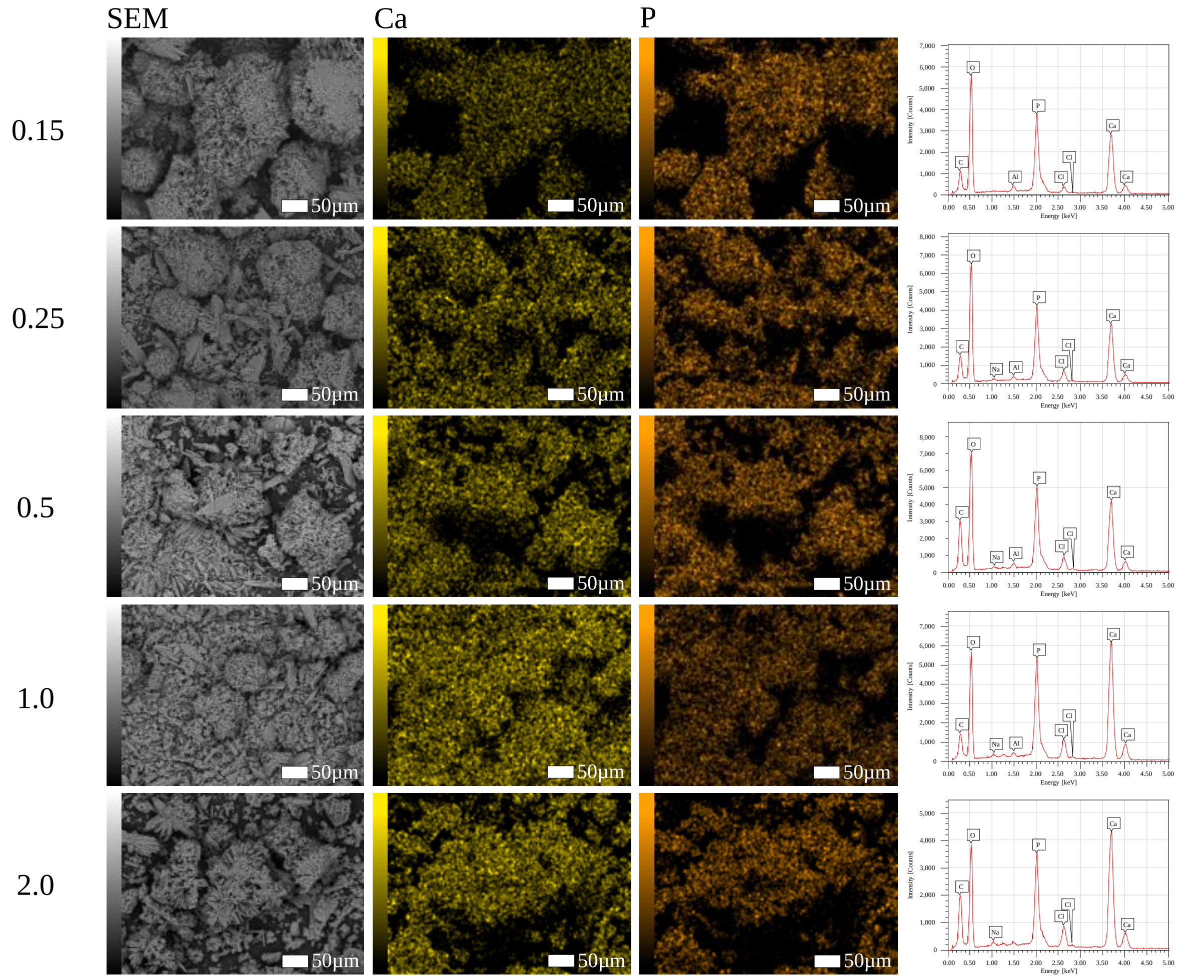

Fig. 3A. *The SEM-EDS analysis of precipitation samples on the 7th day: vertical axis – Urea/Ca^{2+} ratio within samples.*

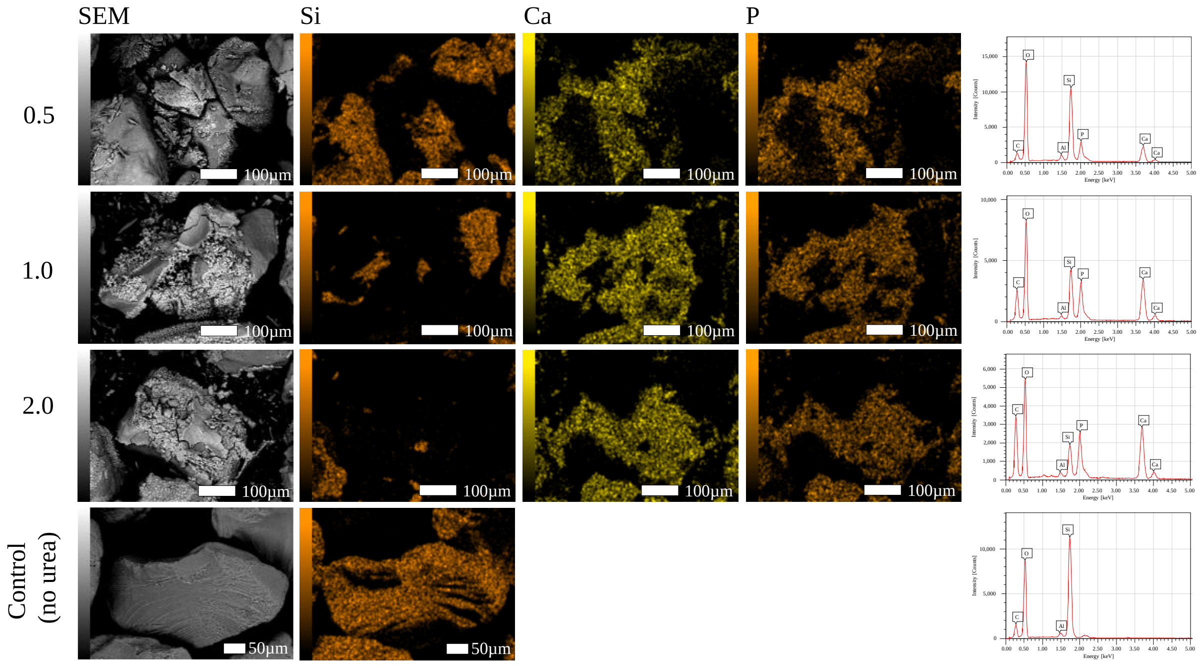

Fig. 4A. *The SEM-EDS analysis of solidification samples: vertical axis – Urea/Ca^{2+} ratio within samples.*
